# Dual pH/redox-responsive hyperbranched polymeric nanocarriers with TME-trigger size shrinkage and charge reversible ability for amplified chemotherapy of breast cancer

**DOI:** 10.1038/s41598-024-57296-4

**Published:** 2024-04-12

**Authors:** Fahimeh Badparvar, Ahmad Poursattar Marjani, Roya Salehi, Fatemeh Ramezani

**Affiliations:** 1 Department of Organic Chemistry, Faculty of Chemistry, Urmia University, Urmia, Iran; 2 Drug Applied Research Center and Department of Medical Nanotechnology, Faculty of Advanced Medical Sciences, Tabriz University of Medical Sciences, Tabriz, Iran; 3 Department of Medical Nanotechnology, School of Advanced Medical Sciences, Tabriz University of Medical Sciences, Tabriz, Iran

**Keywords:** Nano drug delivery systems (NDDSs), pH/redox dual-responsive, Copolymeric nanoparticles, Tumor microenvironment (TME), Biochemistry, Biogeochemistry, Chemistry, Nanoscience and technology

## Abstract

A novel pH/redox-responsive hyperbranched MeO-PEG-b-(NIPAAm-co-PBAE) nanoparticles (NPs) were designed with size shrinkage and charge-reversible potential for targeted delivery of docetaxel (DTX) to MDA-MB-231 cell lines. In the tumor microenvironment (TME), amine protonation induces charge reversal and disulfide bond cleavage under high TME GSH concentration causing size shrinkage, improved deep tumor penetration, and active targeting of the therapeutic agents. These nano drug delivery systems (NDDSs) significantly promoted cancer cell uptake (~ 100% at 0.5 h), facilitating site-specific delivery and deep tumor penetration. The MTT assay revealed significantly higher cytotoxicity (*P* value < 0.0001) for DTX-loaded NPs compared to free DTX. Cell cycle analysis revealed G2/M (58.3 ± 2.1%) and S (21.5 ± 1.3%) arrest for DTX-loaded NPs, while free DTX caused G2/M (67.9 ± 1.1%) and sub-G1 (10.3 ± 0.8%) arrest. DTX-loaded NPs induced higher apoptosis (*P* value < 0.001) in MDA-MB-231 cells (71.5 ± 2.8%) compared to free DTX (42.3 ± 3.1%). Western blotting and RT-PCR assays confirmed significant up-regulation of protein levels and apoptotic genes by DTX-loaded NPs compared to free DTX. In conclusion, TME-responsive charge reversal and size-shrinkable smart NDDSs designed based on low pH, and high glutathione (GSH), offer more effective site-specific delivery of therapeutic agents to tumors.

## Introduction

The development of the chemotherapy process combined with nanotechnology represents a promising treatment approach that considers the complex physiological state of the tumor microenvironment (TME)^1,2^. The TME plays a unique role in tumorigenesis, development, and metastasis^3^. NDDSs are an emerging field for cancer chemotherapy purposes, because of their features to conquer the limitations and numerous drawbacks of conventional chemotherapeutics, such as the lack of selectivity, poor bioavailability, myelosuppression, high dosage of chemotherapeutic drug requirements, adverse side effects, narrow therapeutic index, and multidrug resistance problems by enabling targeted drug delivery to cancer cells^4,5^. Furthermore, in some cases, nanotechnology-based DDSs have already been approved for clinical use^6^. Therefore, fantastic TME-responsive NDDSs with a great distance are considered and exploited for cancer cure, especially as multi-stimuli responsive polymeric nanoparticles that have a significant role in drug delivery systems^7,8^. The nanovehicle destiny inside the biological systems is crucial for biomedical applications^9^. A further understanding of both TME and nano-related physicochemical attributes confirmed that the genetic characteristics of NDDSs, including the shape, size, surface charge, and surface chemical moieties, are hypercritical for the biodistribution, cytotoxicity, and NDDSs' internalization to reach good therapeutic efficacy^10,11^.

Hence, extra- or intracellular signs of TME, including redox (glutathione concentration) and pH lead to fabricating smart stimuli-responsive NDDSs that release chemotherapy drugs in response to specific physiological characteristics in TME, such as low pH or high concentration of GSH^12,13^.

As the most frequent sign of TME targeting, pH triggers charge reversal because of pH differences between TME and the physiological blood environment^14^. Charge-reversal NDDSs (CR-NDDSs) represent a promising platform for developing intelligent NDDSs^15,16^. These innovative NPs have the potential to revolutionize the field of cancer therapy and can turn surface charge from negative to positive in TME^17^. Regular tissue exhibit extracellular pH (pHe) and intracellular pH (pHi) about 7.4 and 7.2, respectively^18^. However, in solid tumors pHe is reduced by 6.5–6.8 or lower, reversing in a reversal in the pHi–pHe gradient via the cell membrane^19^. On the other hand, pH conditions in lysosomes (4.5–5.5) and late endosomes (5.5–6.5), where the nanocarriers are generally located via endocytosis, are considerably less than extracellular pH in tumor tissues.

Moreover, the difference in tumor tissue intracellular concentration of glutathione is one more signal for TME targeting and typically designing size-shrinkable smart NDDSs further to improve tumor-targeting drug delivery^20^. In general, the GSH concentration in the tumor microenvironment is proven to be (5–10) × 10^–3^ M, which is about 2–5 orders higher than that in normal cells (1–5) × 10^−3^ M^21^. Above that, the concentration of GSH in the cytosol was found to be ≈1000 times more than that in the extracellular environment or plasma^22^.

The high GSH concentration in the cytosol accelerates the cleavage of disulfide bonds in the polymeric construction of NPs to free thiols through the redox reaction^23^. This ability can promote the degradation of NPs, thereby allowing the size to shrink and deep penetration^24^.

Docetaxel, is a highly effective anticancer agent, with mechanism action involving disruption of microtubule assembly and cell cycle arrest. However, its limited aqueous solubility, dose-dependent toxicity, and systemic side effects hinder its clinical application. NDDSs could improve DTX's solubility, reduce side effects, and enhance its therapeutic potential to broaden its clinical use^25,26^.

Hyperbranched (Hb) polymers can be mentioned among the most prominent types of polymers that have been utilized to construct diverse drug delivery systems, thanks to the presence of numerous functional terminals, target groups, and drug molecules that can be conjugated to the innumerable functional groups of polymer^27–30^. So that multi-objectives such as targeting groups and chemotherapeutic drugs can be conjugated to Hb polymers simultaneously.

Moreover, poly(b-amino ester)s (PBAEs) are good candidates for engineering CR-NDDSs because of the presence of the tertiary amine groups in its structure with pK_b_ value (~ 6.5) that protonated at weakly acidic conditions^16^.

The atom transfer radical polymerization (ATRP) method is employed to prepare well-defined amphiphilic polymers with complex architecture and controlled polymer molecular weight distribution and degree of branching, which could meaningfully affect the drug encapsulation ratio^31^.

Consequently, pH and GSH have been more interested as particular markers to attain targeted delivery and pH/redox dual-triggered release of therapeutics agents in the tumor cytosol by incorporating protonation/deprotonation of surface groups and cleavage disulfide bonds in the structure of our TME-responsive polymeric nanocarriers with developing of charge-reversal and size shrinkage strategy. It possibly provides robust solutions to convenient the clinical translation of pertinent cancer chemotherapy.

**Figure 1:**
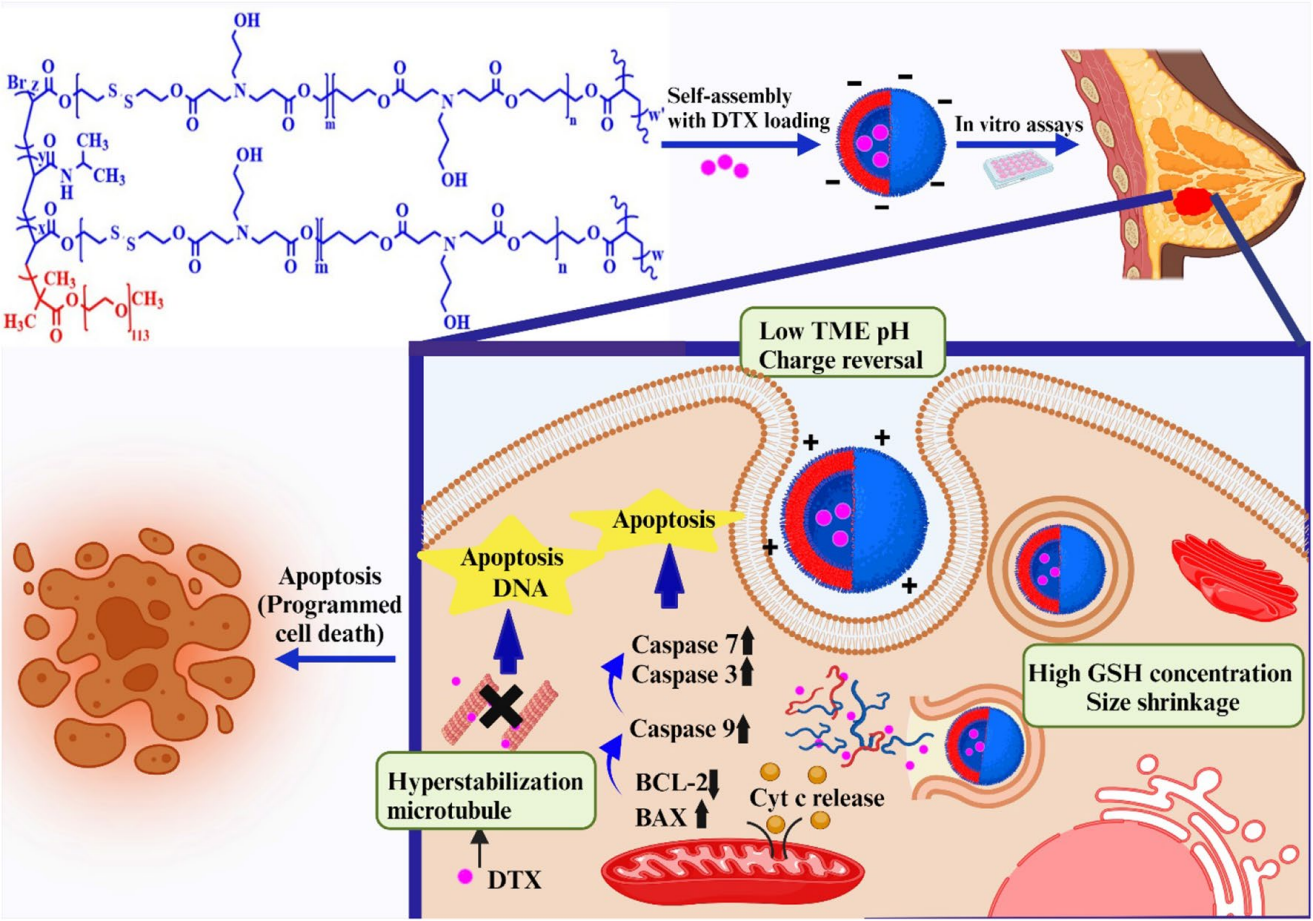
Graphical representation showing the size and charges reversible smart pH/redox dual-stimuli hyperbranched MeO-PEG-b-(NIPAAm-co-PBAE) nano-vehicle for promoted breast cancer tumor targeting and dual pH/redox triggered DTX release. Created with BioRender.com.

The present study synthesized a novel smart pH/redox dual-stimuli responsive Hb NDDSs based on disulfide-containing hyperbranched MeO-PEG-b-(NIPAAm-co-PBAE) polymeric nanoparticles through ATRP. These smart NDDSs could respond to the TME via two dynamic targeting strategies for enhanced tumor penetration, including size-shrinkage and surface charge-switchable behavior. CR-NPs developed here have a negative surface charge in physiological conditions (pH = 7.4) that could avoid recognition by the prolonged circulation time immune system and in the bloodstream. Nevertheless, after reaching TME, it switched its charge to positive due to the protonation at the mild acidic condition of TME. It could interact with the negatively charged cell membrane and enhance cellular uptake of NPs, followed by the burst release of the whole drug in response to high GSH concentration with particle-size shrinkage inside cancer cells (Fig. 1).

## Methods

### Materials and measurements

The MDA-MB-231 (human breast cancer) cell line was acquired from the National Pasteur Institute Cell Bank. Europe GmbH provided penicillin/Streptomycin 100x (Pen/Strep). MTT dye [3-(4,5-dimethylthiazol-2-yl)-2,5-diphenyltetrazolium bromide)-diphenyltetrazolium bromide] gotten from Alfa Aesar, Thermo Fisher Scientific, Heysham. Other biological reagents, including ribonuclease A, rhodamine B (RhB), and propidium iodide (PI), were also obtained from Aldrich. Furthermore, biological materials such as trypsin, RPMI 1640, Trypsin/EDTA, FBS (fetal bovine serum), TRIzol, and were obtained from Gibco BRL. ApoFlowEx FITC as an apoptosis kit was obtained from EXBIO Praha Inc. Deionized water with a resistivity of > 18.2 MΩ was utilized in all experiments. 2 × Master Mix Green, RealQ Plus, and SYBR Green Master Mix were obtained from Ampliqon. Eurofins provided primers).

Other used substances were of analytical grade without further purification. Alfa Aesar supplied stannous octoate Sn(Oct)_2_. Fuchen Chemical Reagents Factory provided sodium iodide (NaI) and 2-mercaptoethanol (MCH). Ethyl acetate (EtOAc), 30% hydrogen peroxide, Sodium hyposulfite (Na_2_S_2_O_3_), and Sodium sulfate (Na_2_SO_4_) were obtained from Beijing Chemical Works. Catalytic 4-dimethylaminopyridine (DMAP), e-Caprolactone (ɛ-CL), polyvinyl alcohol (PVA), CH_3_(OCH_2_CH_2_)_113_-OH (MeO-PEG), trimethylamine (TEA), alpha-bromoisobutyryl bromide (α-BriBr), CuBr, *N*-isopropyl acrylamide, and *N,N,N′,N″,N*″-pentamethyldiethylenetriamine (PMDETA), were obtained from Merck.

All solvents involving diethyl ether (Et_2_O),* N*,*N*-dimethylformamide (DMF), dimethyl sulfoxide (DMSO), methanol, hexane, tetrahydrofuran (THF), K_2_CO_3_, chloroform (CHCl_3_), dichloromethane (CH_2_Cl_2_), isopropanol, acetic acid, 70% ethanol and magnesium sulfate and hydrobromide salt, were purchased from Merck. Docetaxel (Taxotere®) (DTX), acryloyl chloride, 1,4-butanediol diacrylate, 3-amino-1-propanol (AP), and glutathione were bought from Aldrich.

### Copolymer synthesis

#### Synthesis and purification of Bis(2-hydroxyethyl)disulfide (BHES)

To the stirring solution of 2-mercaptoethanol (MCH) (35.1 mmol, 2.74 g) in EtOAc (50 mL) at room temperature, H_2_O_2_ (30%, 35.1 mmol, 1.93 mL) and NaI (0.35 mmol, 52.6 mg), were added for 30 min. Aqueous Na_2_S_2_O_3_ (15 mL) was added to the reaction medium. The obtained product was extracted using EtOAc (9 × 15 mL), dehydrated and dried using Na_2_SO_4_, and the solvent was eliminated. The residue was refined by chromatography (EtOAc: hexane) to generate the pure bis(2-hydroxyethyl)disulfide products. Bruker Spectrospin Avanc ^1^H-NMR (400 MHz) and Bruker Equinox 55 FTIR spectroscopy (Germany) were used for product structural characterization.

#### Synthesis and purification of 2,2′-dithiodiethanol diacrylate (DSDA)

Bis(2-hydroxyethyl)disulfide (17 mmol, 2.57 g) and TEA (136 mmol, 16.7 mL) mixture were prepared by anhydrous THF (50 mL) and kept in an ice bath to reduce its temperature. Acryloyl chloride (68 mmol, 6.04 g) was poured into the chilled compound, then was mixed at 30 °C (1 day). After this step, the THF was eliminated under a vacuum, and the next crude matter was dissolved in chloroform (150 mL). The product was dried using MgSO_4_, and CHCl_3_ was eliminated by rotary evaporation (Lab Tech EV311, Italy). The target product, 2,2′-dithiodiethanol diacrylate (DSDA), was attained as light yellow oil (3.94 g, 90%). FTIR characterized the effects.

#### Synthesis and purification of acrylate-terminated Poly(β-amino ester)s

**Figure 2:**
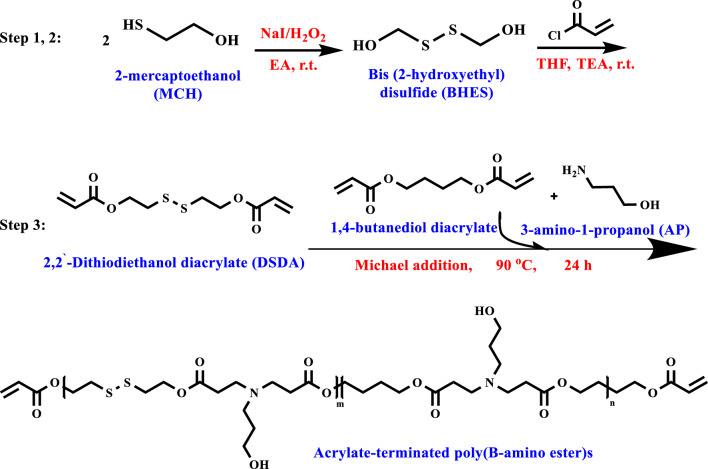
Synthesis route of acrylate-terminated poly(β-amino ester)s through the Michael-type addition from 3 synthetic steps from Bis(2-hydroxyethyl)disulfide (product of step 1) and 2,2′-dithiodiethanol diacrylate (DSDA) (product of step 2).

The polymerization of DSDA and 1,4-butanediol diacrylate (20:80) with 3-amino-1-propanol (AP) is due to the Michael-increasing reaction according to the following typical procedure. Firstly, DSDA (1.2 mmol), 1,4-butanediol diacrylate (4.8 mmol), and dichloromethane (5 mL) were mixed into the dry double-necked round-bottomed flask with a thermometer, a container for condensing, dropping funnel, and stirred for 1 min. Next, AP (5 mmol) diluted with dichloromethane (5 mL) as solvent was poured into the reaction slowly. Then the reaction mixture is placed at the set temperature of 90°Ϲ to be stirred for one day. Then, after the end of the set time for mixing, the prepared product mixture is cooled until it reaches room temperature. Then, after evaporating under a vacuum, it can be used immediately in the subsequent reactions (Fig. 2). ^1^H-NMR and FT-IR endorse the outcomes.

#### Synthesis of bromine-terminal MeO-PEG macroinitiator

**Figure 3:**
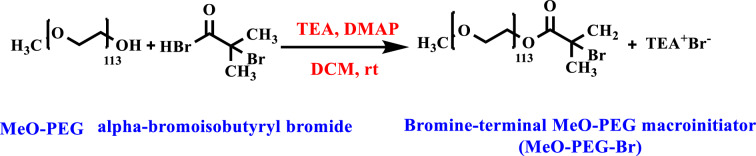
Synthesis route of bromine-terminal MeO-PEG from MeO-PEG and alpha-bromoisobutyryl bromide (α-BriBr) as an ATRP macroinitiator (MeO-PEG-Br).

CH_3_(OCH_2_CH_2_)_113_OH (MeO-PEG) (2.5 mmol, 12.5 g) and CH_2_Cl_2_ (50 mL) were added into a dry-dropping funnel. After the dissolution of MeO-PEG, TEA (5 mmol, 0.7 mL) and DMAP (7.5 mmol, 0.92 g) in CH_2_Cl_2_ (10 mL) were mixed and conveyed into a three-neck round-bottom flask equipped with a dropping funnel, magnetic stirrer, and gas inlet/outlet. Then, it was included in an ice bath (0 °C), and the mixture was degassed through dry nitrogen bubbling. After cooling to 0 °C, α-BriBr (1.54 mL) was added in CH2Cl2 (10 mL). Under continuous stirring, MeO-PEG (2.5 mmol, 12.5 g) was added to the reaction mixture in CH_2_Cl_2_ (50 mL) drop-wise under dry N_2_. Later, the temperature rose to 25 °C and continued under stirring for 18 h. After trimethylamine, hydrobromide salt was eliminated from the reaction mixture by filtration. The solvent was removed, and the concentrated macroinitiator was precipitated in Et_2_O. The cycle was repeated several times before drying in a vacuum oven at 25 °C. Finally, the product was dried, and a white powder of monofunctional bromine-terminal MeO-PEG macroinitiator (MeO-PEG-Br) was obtained. The final product was gained in high yield (94%) (Fig. 3). The ^1^H-NMR and FTIR spectroscopy were used to determine the correctness of the synthesized product.

### Hyperbranched MeO-PEG-b-(NIPAAm-co-PBAE) copolymer synthesized through ATRP approach

**Figure 4:**
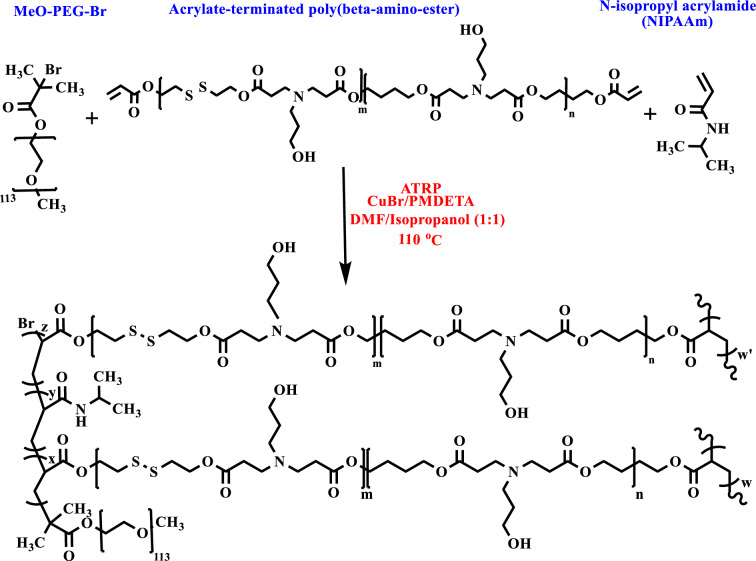
Synthesis route of hyperbranched MeO-PEG-b-(NIPAAm-co-PBAE) copolymer through ATRP approach.

The polymerization reaction was done using the ATRP method, shown in Fig. 4. Inside a three-necked round-bottom flask equipped with a condenser, magnetic stirrer, gas inlet/outlet, DMF (20 mL) and isopropanol (1:1), and MeO-PEG-Br macroinitiator (0.1 mmol, 0.5 g) was also added and stirred 1 h to provide the macroinitiator dispersion. Afterward, PMDETA (0.1 mmol), PBAE (0.2 mmol, 0.48 g), and *N*-isopropyl acrylamide (NIPAAm) (3 mmol, 0.34 g) were added to the dispersion medium, respectively. The mixture was degassed (30 min), and the flask was set under an argon atmosphere. Then, a required amount of CuBr (0.10 mmol, 0.014 g) in DMF (5 mL) was charged into the dry tube separately, sealed with a rubber septum, and deoxygenated three times with three freeze, pump, and thaw cycles using clean argon gas. Next, the deoxygenated CuBr mixture was rapidly added to the reaction medium by syringe. The prepared product in the flask was transferred to a preheated 110 °C oil bath. Then stirred at > 700 rpm, the polymerization process continued for 24 h. After reaction completion (1 day), the reaction medium was cooled to 25 °C and diluted using THF (10 mL). The Al_2_O_3_ column was used to eliminate the catalyst (CuBr). The solvent was removed, and residual traces of unreacted monomers and initiator were separated by dialysis of the crude product mixture using a dialysis bag (cut-off molecular weight 10,000 g/mol). Next, the sample was freeze-dried to obtain a powder. The infrared and ^1^H-NMR spectroscopy determine the structural features and characterization of resulting hyperbranched MeO-PEG-b-(NIPAAm-co-PBAE) copolymers. The ^1^H-NMR and infrared spectroscopy were utilized to specify the structure of the synthesized final product.

### Preparation and characterization of blank and drug-loaded micelle-forming MeO-PEG-b-(NIPAAm-co-PBAE) nanoparticles

Drug-loaded NPs were prepared as follows: Initially, MeO-PEG-b-(NIPAAm-co-PBAE) copolymer and docetaxel were dissolved in 6 mL DMSO at different ratios, 100:10 mg, 100:20 mg, and 100:50 mg. Then, kept under stirring in a light free place for 1 day to let it dissolve completely. The prepared solution was appended to the PVA (0.25%w/v, 20 mL), drop by drop, in an ice bath sonication. DTX-loaded NPs were centrifuged at 4500 rpm for 10 min using Amicon®centrifugal filters. The purified product was lyophilized into powder form and kept at -20 ℃ for further use.

Similarly, for the preparation of the blank NPs, a solution of polymer (100 mg) in DMSO (6 mL) was added gradually to PVA (0.25%w/v, 20 mL) under an ultrasonic probe at a reduced temperature.

The surface charge and size distributions of the NPs were analyzed via DLS. DLS data assessed the colloidal stability of NPs in deionized water and PBS buffer.

The size and morphology of the NPs were further analyzed through FE-SEM–EDX. A UV- Vis determined the encapsulation efficiency (EE) and drug loading content (DLC).

The DLC and DEE are expressed as the following Eqs. (1) and (2):

1 $$DLC \, \left( \% \right) \, = \frac{{\left( {Mass\;of\; drug\; in\; nanocarrier} \right)}}{{\left( {Mass\; of\; nanocarrier} \right)}} \times 100$$

2 $$DEE \, \left( \% \right) \, = \frac{{\left( {Mass \;of\; drug\; in\; nanocarrier} \right)}}{{\left( {Mass\; of\; feed \;drug} \right)}} \times 100$$

Changes in surface charge density of Hb MeO-PEG-b-(NIPAAm-co-PBAE) copolymeric NPs at different pH values were evaluated by identifying their zeta potential alterations after 4 h incubating at pH = 7.4 (physiological conditions) and pH = 6.4 (Tumor microenvironment).

### In vitro dual pH/redox-triggered/controlled DTX release studies of the NPs

For the estimate of DTX release from NPs, the dialysis method (DM) was employed. The drug-loaded NPs (5 mg) were introduced into a dialysis bag containing 2 mL release media (99.5% PBS and 0.5% DMSO) and immersed in a beaker containing 5 mL of four altered release media (pH = 6.4, pH = 6.4 + GSH 10 mM, pH = 7.4, and pH = 7.4 + GSH 10 μM). They were situated in a shaker-incubator at 37 ± 1 °C and 100 rpm throughout the experiment for six days. The release is generally assessed from the outer bulk over time. Samples (5 mL) were withdrawn at definite times (1, 2, 3, 4, 24, 48, 72, 96, and 120 h) and substituted with equal amounts of fresh phosphate buffer^32,33^. At specified time intervals, the DTX content in the samples was analyzed with a UV absorbance of 233 nm. The drug release percentage was estimated using the following Eq. (3):

3 $$Drug \, \;release \, \left( \% \right) \, = \frac{{\mathop \sum \nolimits_{t}^{0} \left( {amount\;of\;drug\;in\;release\;medium\;at\;time\;t} \right) }}{amount\;of\;drug\;loaded\;in\;nanocarrier} \times 100$$

### Kinetic modeling for in-vitro DTX release

Excel software described the most closely correlated release kinetics models of in vitro cumulative DTX release from the engineered nanocarrier. Table 1 presents the mathematical Equations of the models employed to study the mechanism of DTX release from the specific nanocarrier.

**Table 1:** Mathematical models for studying DTX release properties.

Model	Equation
Non-conventional order 2 model	$\frac{1}{{\left( {1 - F} \right)^{{{\text{n}} - 1}} }} - 1 = \left( {{\text{n}} - 1} \right){\text{k}}^{{{\text{n}} - 1}}$ (6)
Hixon-Crowell model	$^{3} \sqrt {{\text{W}}_{0} } =^{3} \sqrt {{\text{W}}_{i} } + {\text{K}}_{{{\text{HC}}}} {\text{t}}$ (7)
Log-Probability model	${\text{Z}} = {\text{Z}}_{0}^{\prime } + {\text{q}}^{\prime } \ln {\text{t}}$ (8)
Peppas (Power Law) model	$f_{1} = \frac{{{\text{M}} _{i} }}{{{\text{M}} \infty }} = {\text{Kt}}^{{\text{n}}}$ (9)

Where W_0_ and W_i_ are the initial and remaining amount of the drug in the system at time t, respectively; ƒ_1_ is the quantity of drug released, M_∞_ is the quantity of drug at the equilibrium state, which may be very similar to the initial amount of drug in the dosage form, M_i_ is the amount of drug released over time t; K_HC_ is the constant of incorporation, which establishes the relationship between surface and volume, K is the quantity of incorporation of structural modifications. F denotes the fraction of drug released up to time t. Z_0_, Z_0_′, q′, n, and k_n-1_ are utilized as the specific values of Z′ when the time is equal to 1. Z and Z′ are probits of the fraction of drug released at time t. Z_0_′ is the values of Z′ when t = 1. The relationships between Z and Z′ with F can be expressed as follows Eqs. (4,5):

4 $$F \, = \left( {2\pi } \right)^{{ - \raise.5ex\hbox{$\scriptstyle 1$}\kern-.1em/ \kern-.15em\lower.25ex\hbox{$\scriptstyle 2$} }} \mathop \int \limits_{ - \infty }^{z} exp\left[ {\frac{{ - Z^{2} }}{2}} \right]dZ$$

5 $$F \, = \left( {2\pi } \right)^{{ - \raise.5ex\hbox{$\scriptstyle 1$}\kern-.1em/ \kern-.15em\lower.25ex\hbox{$\scriptstyle 2$} }} \mathop \int \limits_{ - \infty }^{z\prime } \exp \left[ {\frac{{ - Z^{\prime 2} }}{2}} \right]dZ$$

where *Z* = *(t – t*_*50%*_*)/σ* and *Z*′ = *(logt – logt *_*50%*_*)/σ*′. σ and σ′ are relevant standard deviations^34^.

### Degradation of reduction-responsive disulfidated polymeric NPs triggered by GSH

The The incorporation of a disulfide-bonded crosslinker within the micelle network provides a GSH-sensitive drug delivery that can be cleaved in the presence of high reducing agent concentrations prepared as follows:

The samples (10 mg) were dispersed in PBS buffer with intracellular GSH concentration (pH = 6.4, 10 mM GSH) and then divided into four aliquots. Every aliquot was incubated under their status (0 h of incubation before GSH addition, 0.5 h, 3 h, and one-day incubation at pH = 5.4 with an addition of 10 mM GSH). At predetermined times samples were taken regularly by DLS to follow a change in size distribution.

### Cell culture

The MDA-MB-231 breast cancer cell line was cultured in a T25 for two days before appropriate treatment. The cells were cultured in streptomycin (50 μg/mL), penicillin (1%, 50 IUmL^−1^), and RPMI-1640 medium (10% fetal bovine serum (FBS)) in a CO_2_-filled humidified incubator at 37 °C.

### Cellular uptake of nanoparticles

The qualitative and quantitative cellular uptake of RhB-loaded blank and DTX-loaded Hb polymeric NPs by the MDA-MB-231 cell line was analyzed by Cytation 5 cell imaging multi-mode reader (Bio Tek, USA) and FACScalibur flow cytometer (Becton Dickinson Immunocytometry Systems, San Jose, CA, USA), respectively.

To reach RhB-labeled NPs, the copolymer (10 mg) was dissolved in DMSO (1 mL) and stirred for several hours. Next, the solution and PVA (0.25% w/t, 4 mL) were mixed under probe sonication in a dark condition while having rhodamine B (50 μL of 0.1 mg/mL). To remove the unloaded rhodamine B, the RhB-labeled NPs were filtered by Amicon® centrifugal filters and rinsed several times in dark conditions. The RhB-labeled nanoparticles precipitant was gathered, dispersed in H_2_O (1 mL), and frozen for future use. MDA-MB-231 cells were seeded in 6-well plates at a density of 5 × 10^5^ cells per well and incubated for 48 h. After that, at 70% cell confluence, the medium was replaced with another medium containing 50 μg/mL of RhB-labeled blank and DTX-loaded NPs at 37 °C for 0.5 and 3 h incubation to treatment^35^. Following the incubation time for each group, the cells were trypsinized, rinsed with PBS, and harvested. The intracellular uptake of RhB-NPs was then quantified using the FACScalibur flow cytometer. To simulate different conditions, the uptake of RhB-labeled NPs was evaluated in culture media adjusted to a pH of 7.4 to represent physiological conditions and a pH of 6.5 to mimic the TME.

To assess the effects of DTX-loaded Hb NPs, untreated cells were applied as a negative control group.

### In vitro cell viability assay

In vitro, cytotoxicity of the obtained DTX-loaded MeO-PEG-β-(NIPAAm-co-PBAE) hyperbranched copolymeric nanoparticles and free DTX against the MDA-MB-231 cells were surveyed through the MTT assay method. To reach this aim, MDA-MB-231 cells were cultured in 96-well culture seeds at a density of 10,000 cells/ well in a growth medium (0.2 mL) with FBS (10%) and streptomycin and penicillin (10,000 units/mL) at 37 °C (two days).

After that, the cells acquired almost 70% confluence, and cells received diverse concentrations (20, 10, 5, 2.5, 1.25, 0.625, 0.315, 0.156 μg mL^−1^) of free DTX, and DTX-loaded NPs for two days, then MTT solution (5.0 mg mL^−1^, 30 μL) in PBS was added to each well. By ending the incubation for 4 h, the MTT solution and RPMI medium were aspirated, and DMSO (150 μL) was added to dissolve the formazan crystals. Afterward, the 96-well plate was shaken and recorded at 570 nm via an ELISA plate reader (Awareness Technology, Palm City, FL, USA). All assays were repeated three times. In the end, all tests were carried out by GraphPad.Prism. v9.0.0 P_value_ < 0001.

### Cell cycle analysis

The effect of free DTX, copolymeric micelle, and DTX-loaded NPs on the cell cycle pattern evaluated MDA-MB-231.

MDA-MB-231 cells at a density of 5 × 10^5^ cells/well were seeded in 6-well plates, attached for two days, and then treated with drug-loaded NPs at their IC_50_ doses and free drug for two days. A drug-free NPs culture medium was utilized as a positive control, and the rest of the cells were used as a negative control.

When two days of incubation had passed, the supernatant of the cells was transferred into separate centrifuge round-bottomed tubes; the wells were washed with cold PBS several times, isolated from the plate by trypsin, and conveyed to related round‐bottomed tubes. The supernatant of all the cell-containing tubes was removed after centrifuging. The dispersion of the obtained precipitant was completed with cold and fresh PBS (700 mL), centrifuged again, and then PBS (300 mL) was added to each container containing the centrifuged solution. Precipitated cells were resuspended in PBS (700 µL), and centrifuged. Gently aspirate the supernatant, pour PBS (300 µL) into every cell-containing tube, and resuspend the pellet with careful vortexing. At last, the cell fixation and permeabilization the fixation of cells was done using EtOH (70%, 700 mL) and incubated at 4 °C, three days. After centrifuging, the supernatant of cells was removed to prepare samples for assessment, and PBS (300 mL) was added to each tube. The Ribonuclease A treated cells (10 µL) were stained with propidium iodide solution (10 µL) in the dark. Finally, the FACScalibur flow cytometer was employed to distinguish cell population profiles in various cell cycle phases.

### Apoptosis assay

To verify the impact of free DTX and DTX-loaded MeO-PEG-b-(NIPAAm-co-PBAE) NPs on apoptosis promotion, an apoptosis assay was further assessed by the Ex-bioscience apoptosis detection as reported by the manufacturer’s protocol. MDA-MB-231 cells were seeded in six-well plates at a density of 10^6^ cells per well and incubated for 48 h at 37 °C with 5% CO_2_ to allow for cell attachment and growth. The cells were treated with free DTX-loaded NPs and DTX at their IC_50_ dose at 37 °C for 4 h. After removing the remaining NPs and drugs, fresh full media was added to the cells (48 h). The top materials were collected separately in tubes. The cells were then washed with PBS, and transferred to the related tubes. Additionally, the cells were trypsinized, and centrifuged to remove their supernatants. The cells were then washed twice with PBS. After centrifugation, the top materials were brought out, and the cells were rinsed with an Annexin binding buffer (BB) according to the manufacturer's protocol of the Annexin V staining kit (Exbio). Then, the cells were resuspended in a 100 mL of the binding buffer. Then, 5 mL of Annexin V-FITC (Fluorochrome-conjugated Annexin and 5 mL of propidium iodide (PI) were gently mixed in all samples. The cells were incubated for 15 min in the dark at r.t. After incubation, the cells were centrifuged and resuspended in a binding buffer of 100 mL. The stained cells were then immediately measured using a FACScalibur flow cytometer. The cells treated with blank NPs and Non-treated cells were considered positive and negative controls, respectively.

### RNA extraction and cDNA synthesis for quantitative PCR (qPCR)

MDA-MB-231 cells were plated one day before treatment using the DTX-loaded NPs and free DTX at their IC_50_ value for one day in a 6-well plate at 10^6^ cells/well. The floating of treated and untreated cells was collected and incubated with an utterly fresh medium for two days. The supernatants (plasma) were collected and transferred to their new tubes, and then PBS-rinsed cell pellets were added to the related tubes. After trypsinized, centrifuging was done, and their supernatants were rinsed with PBS twice. Cells lysis and RNA extracted by TRIzol-method. After 2 min incubation at 25 °C, the samples were centrifuged (12,000 g, 4 °C, 20 min), and the RNA phase at the upper aqueous layer was separated. RNA was precipitated through isopropanol (500 μL) addition and centrifuging (20 min, 12,000 g, 4 °C). After rinsing the precipitant with EtOH (75%), it dissolved in DEPC-treated H_2_O. Nanodrop spectrophotometers were employed to determine the RNA content of the solution. Complementary DNA (cDNA) synthesis reaction was accomplished using the Revert Aid RT Kit. All the reactants (cDNA (2 μL), SYBR Green Master Mix (5μL, 2x), deionized water (3 μL), and primer pair mix (5 pmol/μL)) are combined, mixed well, and dispensed in an equal volume for each of the formulations to perform quantitative PCR (qPCR) and analysis apoptotic route.

The PCR program run proceeded at 95 °C for 15 s with 45 cycles of samples after initial denaturation for 15 min. A 50 s annealing/extension step was completed at 60 °C. RT-PCR primer sequences are recorded in Table 2. The non-staining cells were also analyzed as the auto-fluorescence reference. The Real-time RT-PCR information was counted by the gene expression (2^−ΔΔCt^) method, and GAPDH was considered the reference gene for the experiment's analysis.

**Table 2:** Used primers.

Gene	Forward primer (5′–3′)	Reverse primer (5′–3′)
Caspase-3	*GAAATTGTGGAATTGATGCGTGA*	*CTACAACGATCCCCTCTGAAAAA*
Caspase-7	*AGGGACCGAGCTTGATGATG*	*CACTGGGATCTTGTATCGAGGA*
Caspase-9	*CTTCGTTTCTGCGAACTAACAGG*	*GCACCACTGGGGTAAGGTTT*
Bax	*TTCTGACGGCAACTTCAACT*	*CAGCCCATGATGGTTCTGAT*
Bcl-2	*GGGAATCGATCTGGAAATCCTC*	*GGCAACGATCCCATCAATCT*

### Western immunoblotting

Western blotting technique as a gene expression analysis investigated Caspase 7, 3, 9, Bcl-2, Bax, and GAPDH protein levels. To reach this goal, MDA-MB-231 cells (106 cells/well), after being treated with free DTX-loaded NPs and DTX in IC50 doses, were harvested and homogenized with cold RIPA lysis buffer [Tris–HCl (pH = 8, 500 μL], one tablet of protease inhibitor cocktail, NaCl (80 mg), EDTA (3 mg), Triton NP40 (1%, 10 μL), SDS (10 mg), and sodium deoxycholate (25 mg) at 4 °C. Cell lysates were centrifuged (12,000 g, 20 min). The above liquid was collected and analyzed using the Bradford assay and a spectrophotometer for protein determination. After SDS-PAGE separation, the target proteins were transferred to the PVDF membrane (10 μg protein loaded/well). Then blots were blocked with TBST buffer containing 5% (w/v) nonfat dry milk (0.1% v/v Tween®20-tris buffered saline: TBST, and membranes were exposed with antibodies against caspase 3, 9, 7, Bcl-2, Bax and GAPDH at room temperature for 60 min. Increased chemiluminescence was used to visualize Immuno-reactive bands. Amersham®Imager 600 system analyzed all the measurements and was determined using Image J 1.52 n software after normalization to identify protein band intensities. The western blot uses the GAPDH expression as the control.

### Statistical study

Data were presented as mean value ± SD using Microsoft Excel 2019 or Graph pad Prism software (v.9). The ANOVA was utilized as a statistical study for comparing two or more groups. The result was trumpeted as statistically remarkable if the P_value_ was 0.05 or lower.

## Results and discussion

### Polymer design and synthesis

Current work presents a new class of nanoparticles made of amphiphilic biodegradable pH/redox dual-sensitive hyperbranched MeO-PEG-β-(NIPAAm-co-PBAE) copolymer that has been prepared via ATRP (atom transfer radical polymerization) approach for the docetaxel (DTX) delivery in breast cancer treatment. Polymerization has been carried out by growing NIPAAm and our newly designed macromonomer (acrylate-terminated poly(β-amino ester)s) on the MeO-PEG macroinitiator. It should be noted that the reason for choosing the ATRP technique is its ability to control the shape of polymer molecular weight distributions.

A standard protocol for functionalizing acrylate-terminated poly β-amino ester, including disulfide bonds via a selective end-chain modification with special activities, was presented. First, bis(2-hydroxyethyl) disulfide (BHES) dimer has been synthesized by oxidizing two mercaptoethanols with the HOCH_2_CH_2_SH formula by H_2_O_2_. 2,2′-dithiodiethanol diacrylate (DSDA) has been synthesized from BHESS and acryloyl chloride. Then, DSDA and 1,4-butanediol diacrylate (2:8) with AP (3-amino-1-propanol) reacted by Michael-type addition reaction to achieve bis-diacrylate terminated poly β-amino ester (PBAE) including disulfide bond. PBAE acts as a branching macromonomer in synthesizing the final hyperbranched polymer due to two diacrylate groups at both ends of the chain.

### Characterization

#### *FT-IR, and *^*1*^*H-NMR*

FT-IR analysis: The FT-IR spectra of the 2-mercaptoethanol (Fig. S1A), bis(2-hydroxyethyl) disulfide (BHES) (Fig. S1B), 2,2′-dithiodiethanol diacrylate (DSDA) (Fig. S1C), acrylate-terminated poly(β-amino ester)s (Fig. S1D), pure MeO-PEG (Fig. S1E), bromine-terminal MeO-PEG macroinitiator (Fig. S1F), pure NIPAAm (Fig. S1G), and hyperbranched MeO-PEG-β-(NIPAAm-co-PBAE) copolymer (Fig. S1H), associated with detailed explanation were presented in supplementary file.

**Figure 5:**
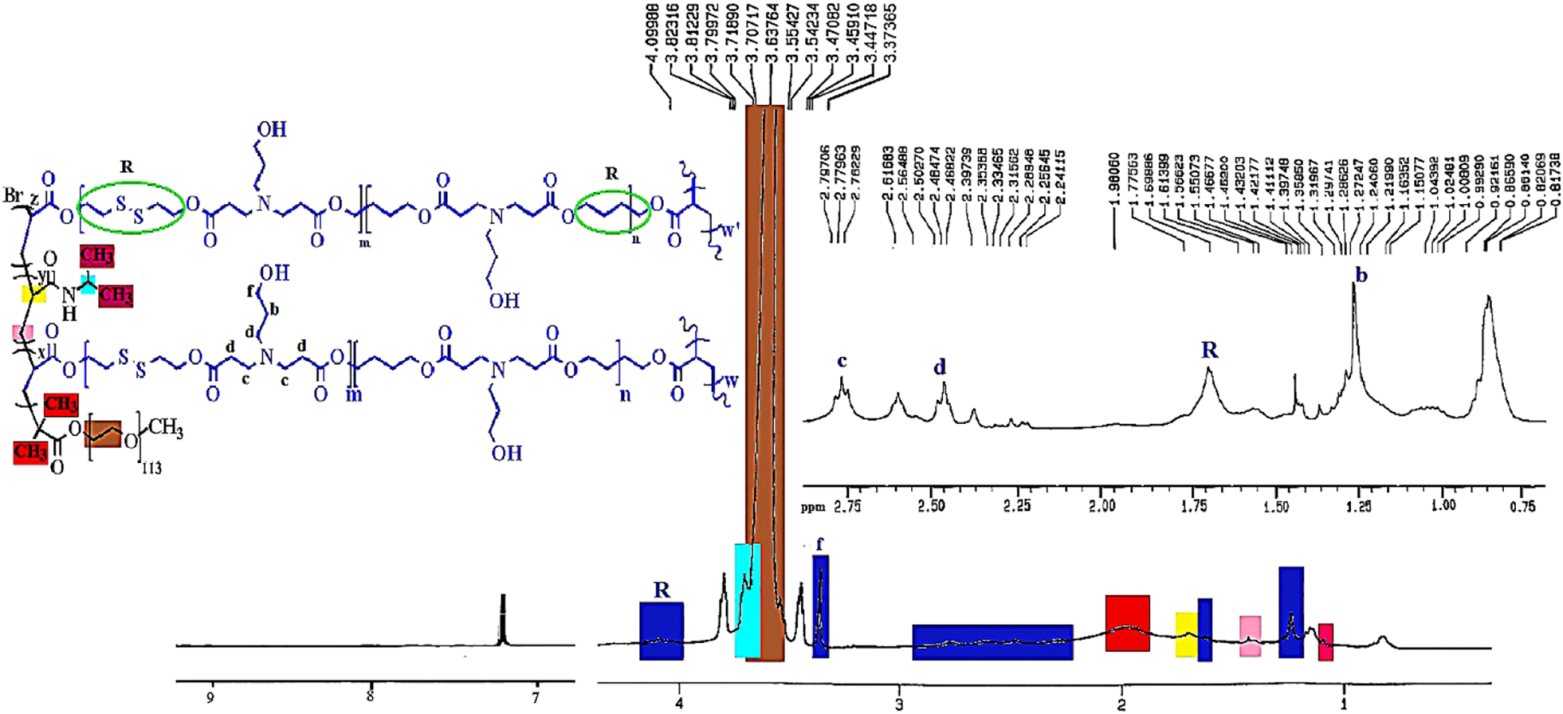
The ^1^H-NMR spectra of MeO-PEG-β-(NIPAAm-co-PBAE) copolymer.

^1^H-NMR Analysis: The detailed chemical structure of products was determined via ^1^H-NMR 400 MHz spectroscopy in CDCl_3_. The ^1^H-NMR spectra related to the synthesized BHES (Fig. S2A), 2,2′-dithiodiethanol diacrylate (DSDA) (Fig. S2B), acrylate-terminated poly (ß-amino ester) (Fig. S2C), MeO-PEG-Br macroinitiator (Fig. S2D), and final MeO-PEG-β-(NIPAAm-co-PBAE) hyperbranched copolymer (Fig. 5) were presented. The detailed explanations were presented in a supplementary file.

### SEM and EDS analysis

**Figure 6:**
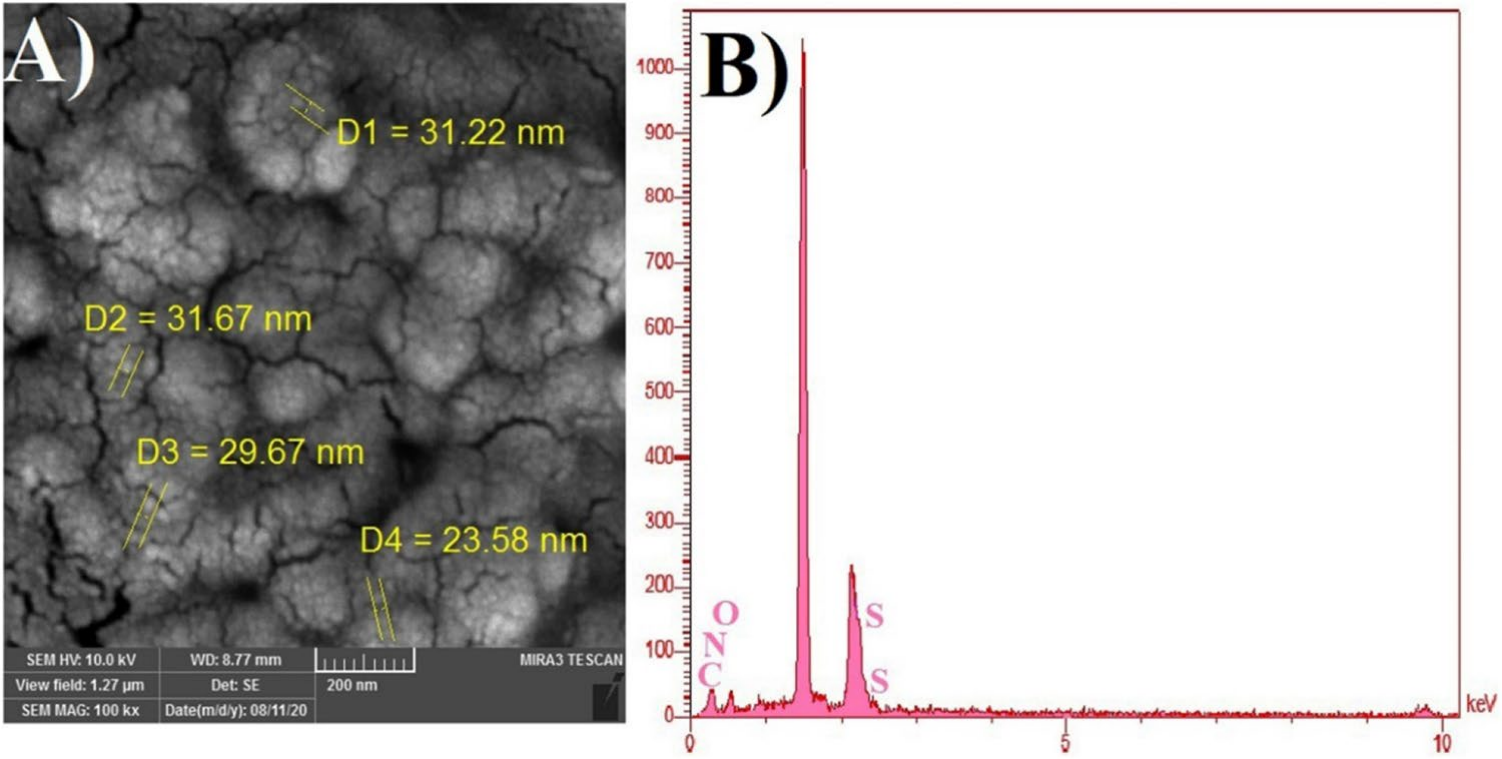
The SEM images (**A**) and EDS analysis of hyperbranched MeO-PEG-b-(NIPAAm-co-PBAE) copolymeric blank NPs (**B**).

SEM–EDS was employed for morphology and size analysis of hyperbranched MeO-PEG-b-(NIPAAm-co-PBAE) copolymeric blank NPs, which were prepared via self-assembly of the copolymer. Semi-spherical NPs have morphology (29 nm), confirmed by the SEM images. Figure 6A indicates an optimal size for passing via the leaky tumor vasculature, sufficiently penetrated in the tumor, and passive targeting.

EDS analysis is performed along with the SEM analysis to determine the chemical composition of the synthesized targeted nanocarriers. As shown in Fig. 6B, the composition mapping indicated the presence of the C, O, N, and S elements. The S element is another evidence for preparing disulfide bond-bridged in the structure of copolymeric NPs. The weight percentage of ingredients is shown in Table 3.

**Table 3:** W% of ingredient.

	C	N	O	S
W%	41.80	14.00	17.43	26.77

### NPs characterization: particles size, zeta potential, colloidal stability and charge-reversible ability

**Figure 7:**
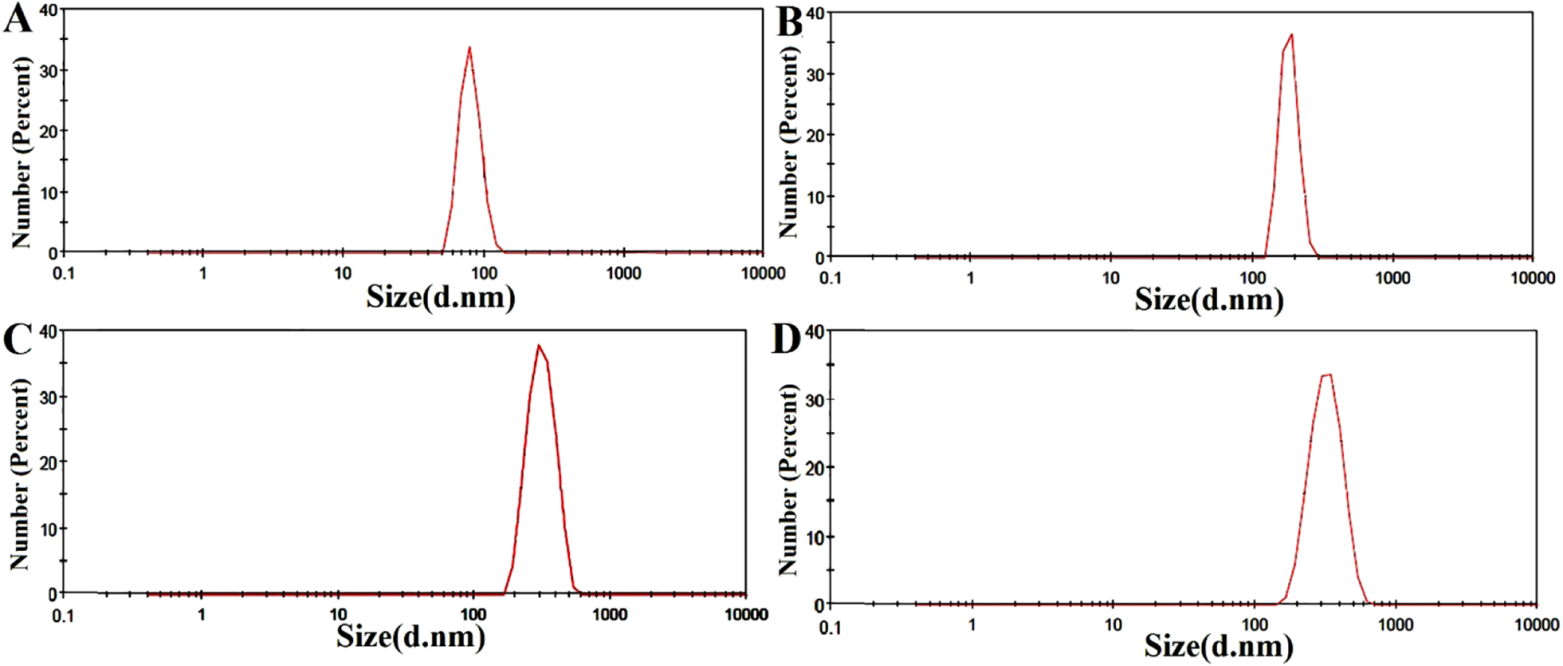
The hydrodynamic size of blank (**A**), and Drug-loaded (**B**) MeO-PEG-b-( NIPAAm-co-PBAE) NPs; The hydrodynamic size of Blank NPs after 1 mount incubation in distilled water (**C**) and PBS buffer (pH = 7.4) (**D**).

The particle size distribution, and zeta potential data of blank NPs acquired by dynamic light scattering are shown in Figs. 7 and S3. The results exhibited that NPs were mono-dispersed with a mean hydrodynamic diameter of 80.24 ± 13.46 nm with a PDI value of 0.4 (Fig. 7A) and a zeta potential of around -10.6 mV (Fig. S3A). Overall, there are differences in the nanoparticle sizes measured by DLS (80 nm) and SEM analysis (29 nm). Preparation methods in DLS and SEM analysis can explain these differences. For DLS assay, nanoparticles are suspended in water and completely hydrated, whereas samples are dried on a surface for SEM. Therefore, a greater diameter was reported for NPs by DLS compared to SEM, which was confirmed by other publications^36^. The range of zeta potential value is ± 20 mV which is considered to achieve stabilization of a nanodispersion. Under this claim, the zeta potential of blank NPs is − 10.6 mV^37^.

After drug loading, according to Figure S3B, the surface charge of NPs observed at + 2.3 mV. Also, NPs size was 80.24 ± 13.46 nm, but after drug loading, it was enhanced to 182.7 ± 26.11 nm (Fig. 7B) with PDI value of 0.6. The particle's size range is between approximately 50 to 200 nm which was approved for passive tumor targeting because of the "enhanced permeability and retention" (EPR) effects. Therefore, hyperbranched MeO-PEG-b-(NIPAAm-co-PBAE) EPR-based NDDSs have the highest tendency to long circulation time and enhanced tumor accumulation^38^.

The colloidal stability of the engineered NPs was evaluated using DLS measurements. After incubating the NPs in distilled water and PBS for one month, their size was determined to be 314.8 ± 70.4 nm (PDI = 0.414), and 323.6 ± 81.29 nm (PDI = 0.414), as depicted in Fig. 7C,D, respectively. It is well-documented in the literature that maintaining colloidal stability with a diameter below 400 nm is crucial for NPs utilized in cancer therapy. This characteristic facilitates the passive targeting of tumors and their leaky vasculature through the EPR effect^39,40^.

The results exhibit robust colloidal stability after a one-month incubation in aqueous and physiological environments. This stability can be attributed to a hydrophilic PEG layer surrounding the NPs, which acts as a steric hindrance to prevent non-specific adsorption and aggregation. These characteristics contribute to enhanced colloidal stability, prolonging the NPs' half-life in the systemic circulation, reticuloendothelial (RES) system clearance, and promoting passive accumulation of the NPs within tumor tissue^41^.

In particular, enhancing the physicochemical surface properties of NPs to improve stability may result in reduced cellular entry. However, this study employed a novel approach to develop stable polymeric NPs that not only maintained stability but also facilitated efficient cellular uptake. This was achieved by using a charge reversal strategy when the NPs were exposed to the TME.

The charge reversal capability of hyperbranched MeO-PEG-b-(NIPAAm-co-PBAE) nanoparticles was assessed by detecting their zeta potential after 4 h incubation under weakly acidic conditions with a pH of 6.4. As depicted in Figure S3C, the overall zeta potential of the nanoparticles increased from − 10.6 to + 7.46 mV. In the bloodstream's neutral pH environment (~ 7.4), the engineered NDDSs carry a net negative charge. In contrast, these polymeric NPs undergo a charge reversal phenomenon, transitioning to a positive charge upon reaching the lower pH environment of the tumor tissue (~ 6.4). The charge reversal ability of these NPs should belong to the protonation of tertiary amine groups at weakly acidic TME^42^.

According to the results, engineered MeO-PEG-b-(NIPAAm-co-PBAE) NPs could sustain their original negatively charged status in the bloodstream and hastily transfer into the positively charged status once the acidic endo/lysosome is entered following cell internalization by endocytosis. Meanwhile, according to some studies, the charge-reversal strategy can accelerate endosomal escape^43^. So, our CR-NDDSs may be favorable for the endo/lysosomal escape and release of their cargoes in cancer cells.

NDDSs negatively charged relevance could contribute more to the lengthening blood circulation time than the positively charged NPs with similar particle size^44^.

Zhang et al. reported pH and redox dual-responsive nanoparticles with the charge-reversible ability to co-deliver gene and chemotherapeutic agents. The responsivity of NPs owned to the rupture of the β-carboxylic amide bond under the weakly acidic status. So, CAPL/ssPBAE-MTX/pDNA NPs kept their original negatively charged status in the bloodstream but rapidly changed into the positively charged status once the acidic endo/lysosomes were entered following cell internalization by endocytosis^45^. A study by Li et al., charge-reversal NDDSs for co-delivery of vascular endothelial growth factor siRNA and etoposide for metastatic non-small cell lung cancer treatment^46^. A negatively charged shell is provided via histidine moieties, improving the system's blood circulation stability. The acidic microenvironment triggered the protonation of imidazole groups in histidine following the EPR effect-mediated tumor accumulation, resulting in the charge-reversal from negative to positive, which enhanced deep tumor penetration and cell internalization. According to studies in this field^47^, and the results of this test, hyperbranched MeO-PEG-b-(NIPAAm-co-PBAE) NPs are suitable candidates in CR-NDDSs.

### Entrapment efficiency

Nanocarriers entrapment efficiency (EE) is crucial in developing nanoparticle delivery systems^48^. The MeO-PEG-b-(NIPAAm-co-PBAE) copolymeric NPs exhibited a drug encapsulation efficiency (DEE) of 98.3% and a drug loading capacity (DLC) of 9.83% for the 10:1 ratio of nanoparticles to drug. This high DEE and DLC value indicates that the new copolymeric NPs possess a remarkably high drug-loading capacity. Additional DEE and DLC values can be found in Table S1.

### pH/Redox-induced size change of the nanoparticles

**Figure 8:**
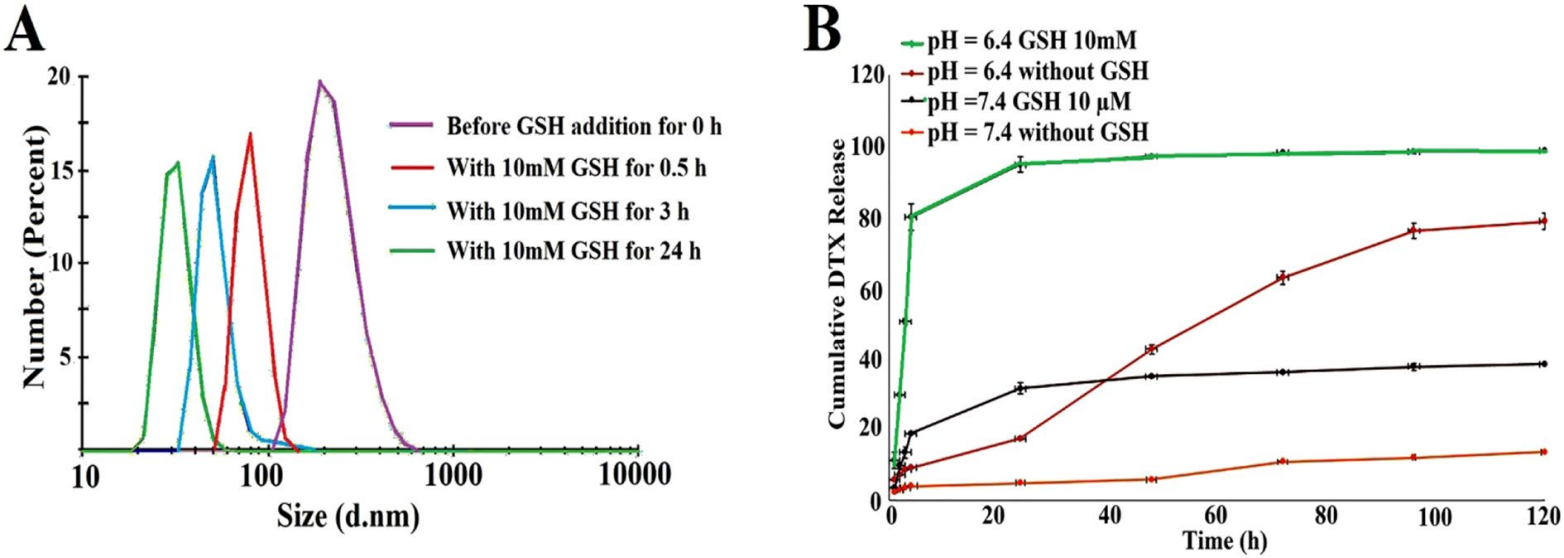
In vitro, dual pH/redox triggered DTX release from MeO-PEG-b-(NIPAAm-co-PBAE) NPs at pH = 6.4 with 10 mM GSH (green), pH = 6.4, without GSH (red), pH = 7.4 with 10 µM GSH (black), pH = 7.4 without GSH (orange) (**A**). pH/redox-responsive size alteration assessment at PBS buffer with a pH of 7.4 without GSH and PBS buffer with 10 Mm GSH and pH of 6.4 at different time intervals (0.5, 3, and 24 h) (**B**).

The redox-responsively of disulfide bond-bridged MeO-PEG-b-( NIPAAm-co-PBAE) NPs was approved by measuring the size changes in response to 10 mM GSH in PBS buffer (pH = 6.4) medium by DLS measurement which was another evidence for the successful incorporation of disulfide bonds within the nanoparticle network. Remarkably, the size reduction of the reducible SS-NP NPs was observed in definite time intervals (0.5, 3, 24 h). As illustrated in Fig. 8A, the average size of nanoparticles before the GSH addition in PBS buffer with a pH of 7.4 was around 80.24 ± 13.46 (PDI = 0.4) nm. The average size of SS-NPs increased to 227.0 ± 73.6 (PDI = 0.8) nm after being incubated in 10 mM GSH and at pH 6.4 for 0.5 h, indicating quick disassembly of SS-NPs. It reaches a diameter of around 92.48 ± 16.28 (PDI = 0.7) nm in 3 h. The significant NPs size change decreased to 32.10 ± 5.9 (PDI = 0.6) nm after exposure to 10 mM GSH at pH 6.4 for 24 h. The millimolar level of GSH can fragment the disulfide bonds and lead to a significant change in nanoparticle size. It can be concluded that the size change of NPs depends on the rupture of disulfide bonds in response to the intracellular reductive microenvironment and protonation of amine groups of PBAE in slightly acidic TME pH.

### In vitro pH/GSH dual-triggered DTX release study

In general, the pH values of tumoral lysosomes (pH = 4.5–5.0) and endosomes (pH = 5.0–6.5) are both lower than those (pH = 7.4) in blood circulation and normal cells. GSH level is increased to higher levels in cancerous tissue because glutathione (GSH) content is 1000 times higher in cancer cells than normal cells^49^. Therefore, pH/redox-dual responsive delivery vehicle can effectively facilitate intracellular delivery of encapsulated anticancer drugs in tumor tissue.

pH/redox responsive behavior of synthesized MeO-PEG-b-(NIPAAm-co-PBAE) NPs was evaluated in response to pH and GSH in various release media, involving, (i) in PBS with pH = 6.4, (ii) in PBS with pH = 6.4 and 10 mM GSH, (iii) in PBS with pH = 7.4 and 10 μM GSH, and (iv) in PBS with pH = 7.4. All release systems were kept at 37 °C under continuous stirring. The high dependency of DTX release pattern from engineered smart NDDSs in response to the pH and GSH alteration in a time-dependent manner was evaluated in Fig. 8B. The release medium of pH = 7.4 + GSH 10 μM exhibited about 31% DTX release in one day, while DTX release at pH = 6.4 + GSH 10 mM media was up to 95% simultaneously. The biodegradable hyperbranched MeO-PEG-b-(NIPAAm-co-PBAE) copolymeric NPs released 100% of its content in tumor microenvironment conditions (pH = 6.4, GSH 10 mM). This dominant GSH-responsive DTX release returns to disulfide bond-bridged hyper branch block scission. The pH-responsive behavior of DTX release in GSH absent medium becomes more prominent with time. Also, after one day, DTX release at pH = 6.4 and pH = 7.4 without GSH reached 17% and 4%, but after 120 h, it got 80 and 10%, respectively. Therefore, MeO-PEG-b-(NIPAAm-co-PBAE) Hb NDDSs were more sensitive to pH and GSH concentration.

### Kinetic studies

The experimental data were checked using four kinetic models, and the highest correlation model for the release of DTX from the nanoparticles was identified and listed in Table 4. The appropriate model for drug release kinetics was determined based on the Maximum R-squared (RSQ) values obtained and the minimum Mean Percent Error (MPE) calculated for each release curve.

**Table 4:** Fitting parameters of the in vitro DTX release data to different release kinetics models.

Release media	pH = 7.4,GSH = 0	pH = 6.4, GSH = 0	pH = 7.4,GSH = 10 µM	pH = 6.4, GSH = 10 mM
Maximum RSQ	0.970	0.984	0.914	1.000
Minimum MPE	10.648	8.872	18.893	1.156
Proposed model	Non-conventional order 2	Hixon-Crowell	Log-Probability	Peppas (Power Law)

### Cellular uptake of DTX-loaded nanoparticles

To study the cellular uptake capacity of DTX-loaded MeO-PEG-b-(NIPAAm-co-PBAE) NPs and blank NPs in a time-dependent manner, nanocarriers were labeled with widely used rhodamine-B fluorescent marker and the quantitative internalization of nanocarriers in the MDA-MB-231 cells was surveyed for 0.5 h and 3 h. The detected fluorescence intensity was directly related to the amount of rhodamine-B labeled nanocarriers internalized. As was seen in Fig. 10, a Cytation 5 cell imaging multi-mode reader was conducted to assess intracellular uptake qualitatively. The rhodamine-B labeled DTX-loaded MeO-PEG-b-(NIPAAm-co-PBAE) NPs were mainly evaluated in the cytoplasm, showing a swift uptake of rhodamine-B labeled samples into MDA-MB-231 cells.

**Figure 9:**
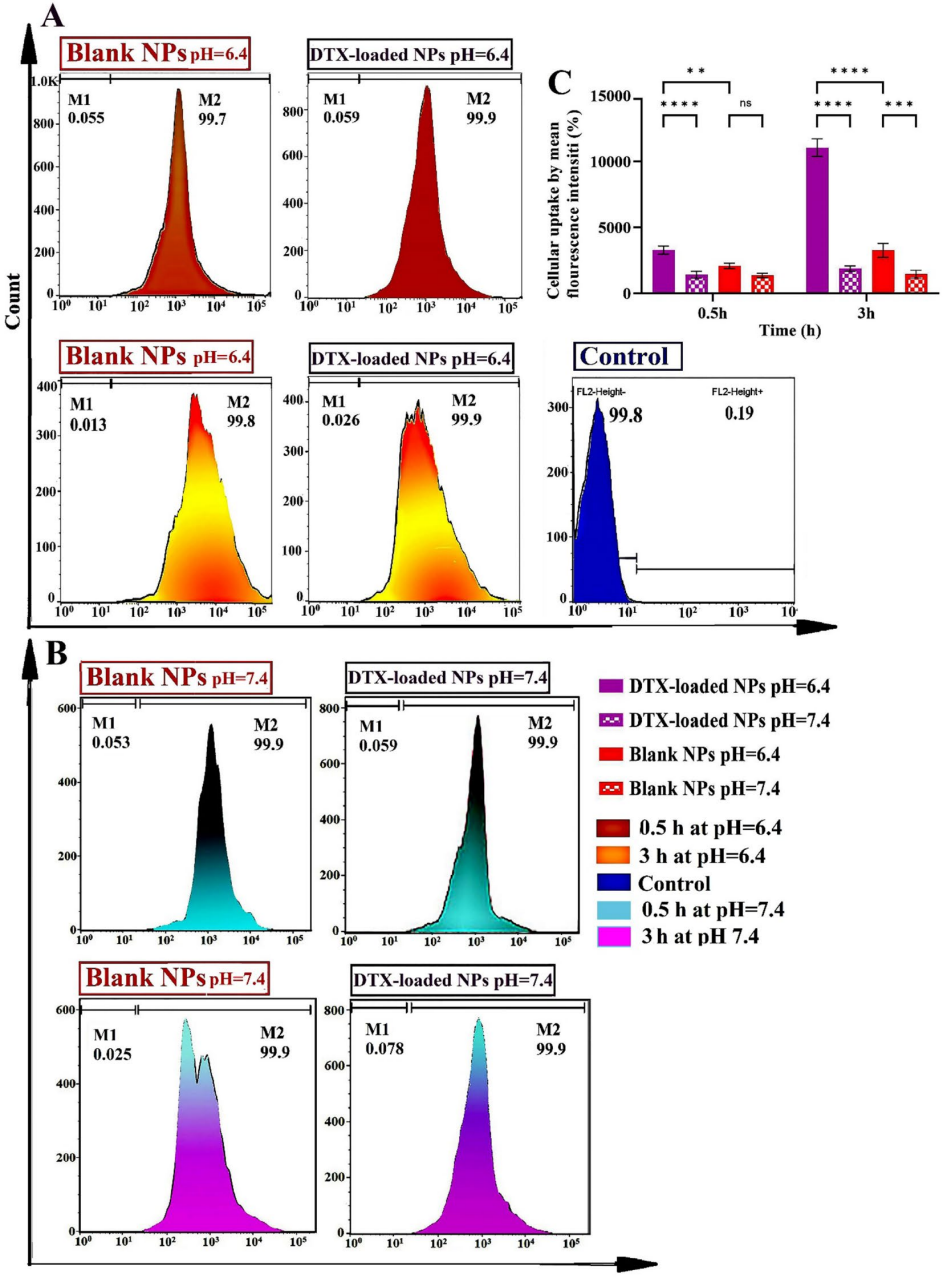
Flow cytometry diagrams for intracellular uptake indicating quantitatively examine fluorescence intensity at pH = 6.4 (**A**), and pH = 7.4 (**B**); And comparison graphs of mean fluorescence intensity (%) of intracellular uptake at pH 7.4 and 6.4 (*P*_value_ ˂0.0001) (**C**) of MDA-MB-231 cells after treatment with RhB-labeled DTX-loaded MeO-PEG-b-(NIPAAm-co-PBAE) NPs and, RhB-labeled copolymeric Blank NPs in various time intervals: 0.5, and 3 h.

**Figure 10:**
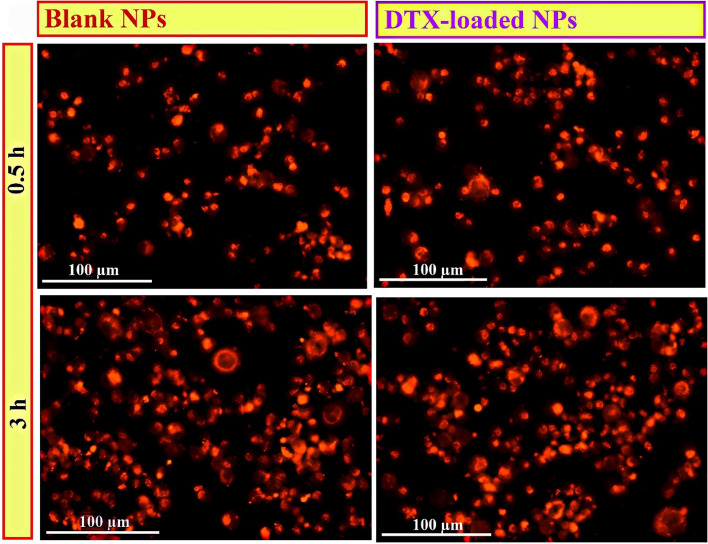
Qualitative image by Cytation 5 cell imaging of MDA-MB-231 cells after treatment with RhB-labeled DTX-loaded MeO-PEG-b-(NIPAAm-co-PBAE) NPs and, RhB-labeled copolymeric NPs in various time intervals: 0.5, and 3 h at TME.

The quantitative cellular uptake measurements have been shown in Fig. 9A, resulting in ~ 100% of gated cells Rh-B positive at all predetermined periods. By increasing the exposure time from 0.5 to 3 h, the mean fluorescent intensity in MDA-MB-231 cells treated with drug-loaded NPs enlarged from 3266 ± 310 to 11,042 ± 669 and blank NPs increased from 2075 ± 206 to 3244 ± 531 (Fig. 9C). The higher cellular uptake of RhB-labeled drug-loaded NPs compared to blank NPs is caused by switching of the surface charge after drug loading from negative (-10.6 mV) to positive (+ 2.3 mV). Because of less electrostatic repulsion forces of positive charge with the negative cell membrane, it leads to higher uptake into the cells. The outcomes showed that rhodamine B-labeled nanocarrier accumulation in the cells after treatment has a rapid and significant uptake, and it is validated by our cellular uptake study by citation 5 multimode reader. Off note, at the physiological normal pH range (7.35–7.45), most proteins in the blood have an isoelectric point of about ~ 4.7, which means that most proteins like human serum albumin (HA) in the blood have a negative charge^50^. Thus nano-vehicle's positively charged forms would effectively associate with the harmful proteins. Therefore, electrostatic interactions between the cytomembrane and the positively charged nanocarriers are essential for cellular uptake. Hence, it is concluded that after switching the surface charge of NPs from negative to positive exposure to TME, negatively charged cell membranes attract them, facilitating cellular uptake. At flow cytometry analysis, untreated cells were considered a control group. These outcomes are consistent with enhanced cell uptaken results through the positive charge of the nanoprobe-promoted electrostatic interactions with cell membranes in an acidic environment reported by Chen et al.^51^. Comparison of the cellular uptake of blank and DTX-loaded NPs at pH 6.4, and 7.4 shows that the cellular uptake of NPs at pH 6.4 is considerably higher than that at pH 7.4 (*P*_value_ < 0.0001), indicating the pH-triggered enhancement of cellular uptake at TME (Fig. 9B). The mean fluorescent intensity value of blank and DTX-loaded NPs at pH 7.4, was 1341 (0.5 h), 1446 (3 h) and 1392 (0.5 h), 1865 (3 h), respectively (Fig. 9C). The mechanism of facilitating internalization of the NPs can be described as follows: Initially, within the acidic TME, the NPs surface charge undergoes a switch to a positive charge (to + 7.46 mV) due to the amine groups of PBAEs chains. This alternation in surface charge facilitates a higher cellular uptake of the NPs by cell due to the weakened electrostatic repulsion forces between the NPs and the negatively charged cell membrane^16^. Additionally, increased concentration of GSH in tumor tissues triggers the size shrinkage response in the NPs, due to the rupture of disulfide bonds in copolymeric structure, causing them to shrink in size. Consequently, the engineered NPs exhibit a rapid and substantial uptake rate, indicating the ability of NPs to adjust their charge and size sensitivity in response to the TME stimuli, thereby enhancing cell internalization (Fig. 10).

### In vitro cytotoxicity studies free and DTX-loaded NPs against MDA-MB-231 cell line

**Figure 11:**
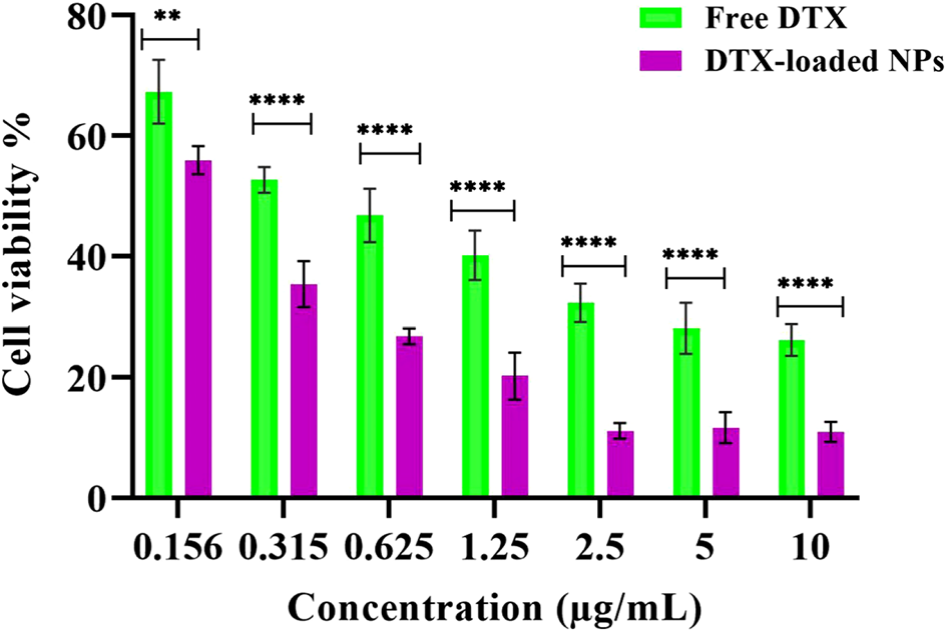
The viability of MDA-MB-231 cells incubated with different concentrations of free DTX and DTX-loaded NPs for 48 h was determined by the MTT method. Data are presented as the average ± SD (n = 6) (*P*_value_ < 0.0001).

Docetaxel has a primary practical tumor-suppressive function and plays an efficient role in inhibiting cancer cell proliferation. In vitro cellular cytotoxicity activities of the DTX-loaded MeO-PEG-b-(NIPAAm-co-PBAE) NPs and free DTX (Fig. 11) against MDA-MB-231 breast cancer cell lines for two days and their inhibition concentration (IC_50_) was indicated. Lower viability in the case of DTX-loaded NPs (IC_50_ = 0.20 ± 0.02 µg mL^−1^) compared to free DTX (IC_50_ = 0.3441 ± 0.04 µg mL^−1^) may correspond to their efficient cellular uptake and drug release leading to increased drug availability inside the tumor cell. The in vitro cytotoxicity results illustrated that the DTX-encapsulated dual pH/redox-responsive MeO-PEG-b-(NIPAAm-co-PBAE) copolymer, in contrast to the free DTX, could foster toxicity at all tested concentrations against MDA-MB-231 cells, which implied GSH and pH-dependent DTX release within the tumor and contributed to intense cytotoxicity. Also, MDA-MB-231 cells were incubated with a serial concentration of blank NPs from 1.56 to 100 µg mL^−1^, and the cell viability in all concentrations was above 90%. Therefore, blank NPs did not have an impact on cytotoxicity tests that were done in this cell line.

Shweta Arora et al. studied the toxicity of docetaxel multi-walled carbon nanotube conjugates in breast cancer cells and demonstrated increased cytotoxicity compared to free DTX^52^. Another study revealed that cRGD-conjugated NPs (i.e., H40-DOX-cRGD) showed high cellular uptake in human glioblastoma (U87MG) cells for tumor-targeted drug delivery^53^.

### Apoptosis assays

#### Cell cycle arrest

**Figure 12:**
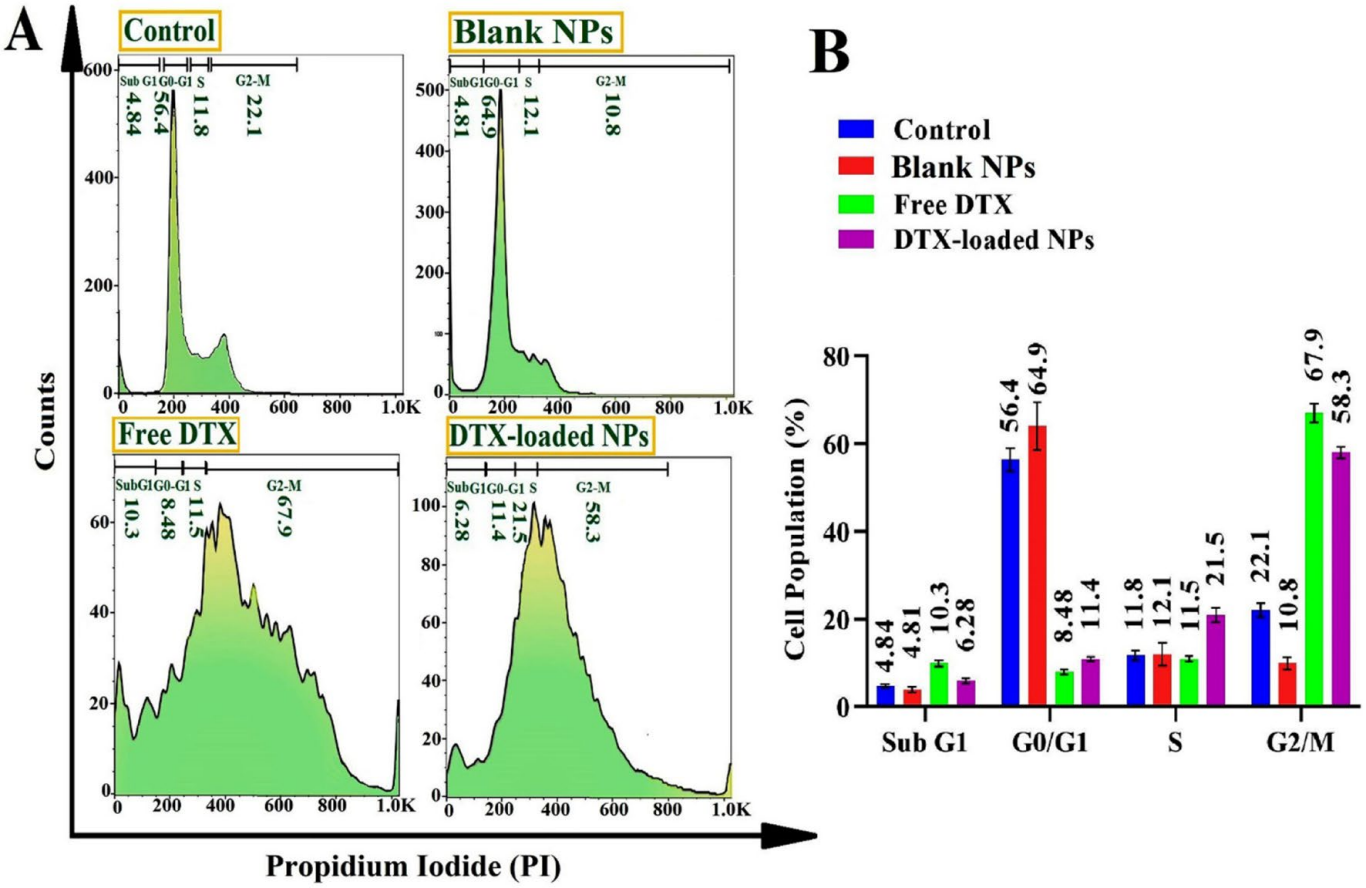
Cellular interaction for cell cycle analysis of all formulation (DTX-loaded NPs, free DTX, blank NPs, and control cells) with MDA-MB-231 cells, by flow cytometry technique: Histograms of cell cycle distribution (**A**) and quantitative diagram of the related flow-cytometry diagram's cell population (%) profile in cell cycle phases (Sub G1, G2/M, S, and G0/G1) (**B**).

The cell cycle is the sequence of four events (G1, G2, M, and S) in a cell. The cell cycle analysis as an essential mechanism during nanoparticles-induced cytotoxicity can be used to assess a stopping point in the cell cycle called cell cycle arrest, where it is no longer involved in the duplication and division processes. The effect of DTX-loaded MeO-PEG-b-(NIPAAm-co-PBAE) NPs, free NPs, and free DTX on the cell cycle was studied to characterize DNA content in cells utilizing propidium iodide is illustrated in Fig. 12.

Upon exposure of MDA-MB-231 cells to DTX-loaded NPs, and free DTX compared to the control group, there was a sharp rise in the cell's proportion in the G2/M phase cell cycle from 22.1 to 67.9% and 58.3%, respectively.

The results indicated that a significant G2/M arrest checkpoint permitted cells to enter the mitosis phase, leading to cell apoptosis following treatment with DTX-loaded NPs and free DTX. The higher in vitro anti-tumor effect could be attributed to the intelligent pH/redox-triggered controlled release of DTX in TME. DTX-loaded NPs induce higher levels of G2/M (58.3 ± 2.1%) and S (21.5 ± 1.3%) phase arrest in IC_50_ dosage owing to the intracellular uptake and rising solubility of DTX-loaded NPs, while free DTX treatment groups induces G2/M (67.9 ± 1.1%) and Sub G1(10.3 ± 0.8%) arrest.

Ghassami et al. research indicated that the cell cycle was blocked in the G2/M phase using Apt-DTX-loaded NPs and free DTX following treatment and did not alter the anti-microtubule mechanism^54^. Chen and co-workers studied G2/M phase arrest using anti-(prostate-specific membrane antigen) (PSMA) aptamer-decorated DTX-loaded NPs against the LNCap prostatic cancer cells^55^.

Cell cycle results illustrated higher cell arrests for DTX-loaded NPs, consequently showing notable apoptotic induction due to the enhanced cellular uptake^56^.

### Quantitative analysis of apoptotic cell death: annexin V/PI staining method

**Figure 13:**
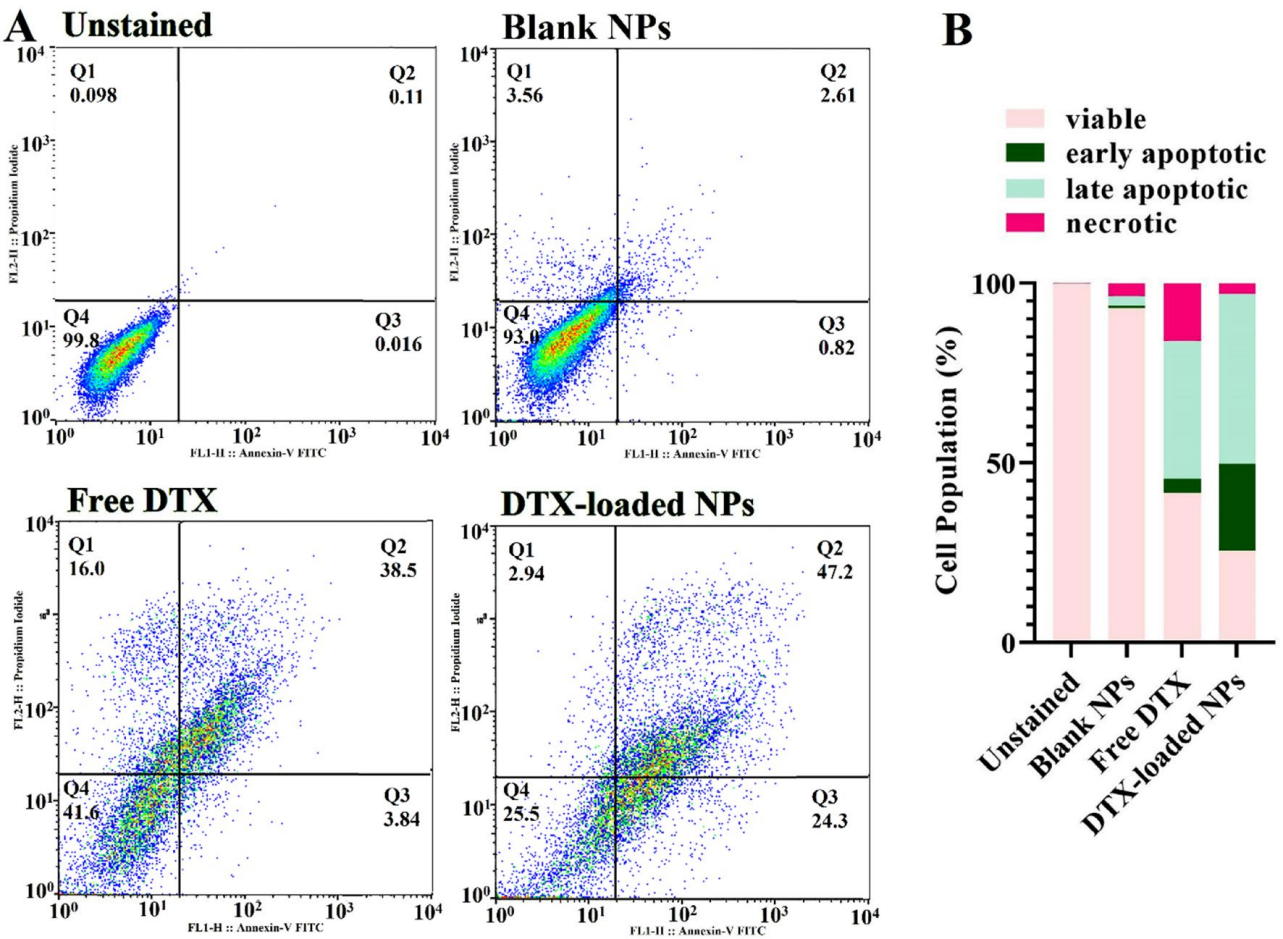
Cytogram of cellular apoptosis against MDA-MB-231 cells induced by free DTX and DTX-loaded NPs in their IC_50_ dose in comparison to the blank hyperbranched MeO-PEG-b-(NIPAAm-co-PBAE) copolymeric NPs as positive control and non-treated cells as a negative control. The flow cytometric diagram divided cells into four sections (viable, early apoptotic, late apoptotic, and necrotic) and the percent of counted cells in each area is illustrated on the quadrant charts (*P*_value_ < 0.001) (**A**). Quantitative results of apoptotic effects evaluated by Annexin V/FITC assay (**B**).

To survey the proportion of cell death by apoptosis or necrosis in MDA-MB-231 cells treated with free DTX, blank NPs, and DTX-loaded NPs, an Annexin V assay was done. As shown in Fig. 13, after treatment with an IC_50_ dose of DTX alone and DTX-loaded NPs, the population of all early and late apoptotic cells was 42.34 ± 3.1 and 71.5 ± 2.8% (*P*_value_ < 0.001), respectively. It is observable that DTX-loaded NPs could increase the percentage of total apoptosis by 1.68-fold compared with free DTX in MDA-MB-231 cells. The outcomes revealed that blank NPs didn't show noticeable apoptosis (2.43 ± 1.2%) and necrosis (3.56 ± 1.3%). Therefore, the engineered NPs are non-toxic for MDA-MB-231 cells. These results indicate that DTX-loaded NPs induced more programmed cell death through apoptosis than the free DTX. The more significant apoptosis proportion was due to the charge reversal ability of engineered NPs from negative in the bloodstream (pH = 7.4) to positive status in TME (pH = 6.4), which led to interaction with the negatively charged cell membrane and enhanced cellular uptake of NPs. On the other hand, because of GSH-responsive DTX release from the NPs and their size shrinkage in TME, higher cytotoxicity, and apoptosis were observed in DTX-loaded NPs groups compared to free DTX. These results confirmed the cell cycle data, where the DTX-loaded NPs induced apoptosis. In a study by Zhao et al. A2780/T cells were incubated with DTX-loaded PBAE NPs and free DTX for 24 h. Their results show that the percentage of apoptosis in cells treated with free DTX and DTX-loaded PBAE NPs was 23.5 and 44%. Interestingly, their cell cycle results showed that free DTX and DTX-loaded NPs arrested cells in G2/M (21.2%) and S and G2/M, respectively, confirmed our finding^57^.

### Investigation of cell apoptosis by real-time PCR over treatment of MDA-MB-231 cells via docetaxel-loaded hyperbranched MeO-PEG-b-(NIPAAm-co-PBAE) NPs

To investigate the apoptosis pathway as a form of programmed cell death, caspase-dependent pathways were analyzed by Real-Time PCR. Caspase (cysteine-aspartic proteases) plays a central role in the apoptosis network, which has been classified into two categories: Executioner caspases (7, 6, and 3) and initiator caspases (9 and 8). Also, the pro-apoptotic Bax gene initiated cytochrome c release and Bcl-2-regulated apoptosis.

**Figure 14:**
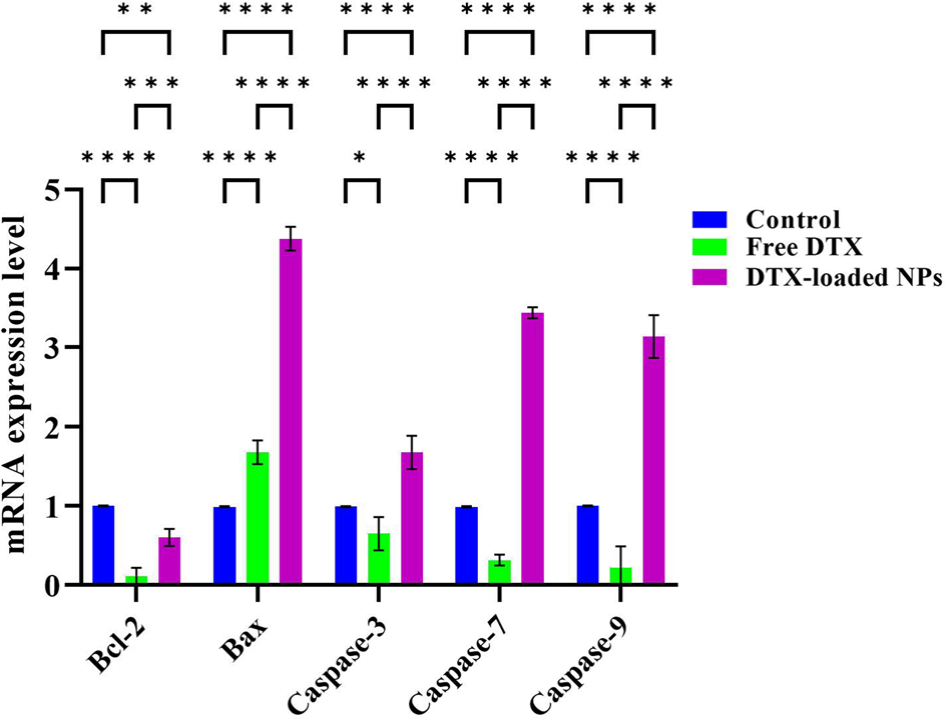
Results of apoptotic effect of free DTX and DTX-loaded NPs treated MDA-MB-231 cells assessed by real-time PCR analyses at the gene level. Untreated cells are considered as control. (*P*_value_ < 0.0001).

Figure 14 presented the results of the Real-Time PCR experiment in gene expression for cells treated with free DTX and DTX-loaded NPs versus the control group. The Bcl-2 gene was down-regulated in the free DTX and DTX-loaded NPs treated groups, which agrees with apoptosis. Also, as expected, Bax was up-regulated in both cases, leading to the release of cytochrome-c by the mitochondria into the cytosol and binding to and activating Apaf-1. Of note, the Bax upregulation level in the DTX-loaded nanoformulation group is more significant than free DTX, leading to the activation of caspase 9. The caspase 9 activation became more up-regulated in the DTX-loaded hyperbranched MeO-PEG-b-(NIPAAm-co-PBAE) NPs versus the free DTX. Active caspase 9 then activates the common apoptosis pathway genes of caspases 7 and 3 in treated cells by DTX-loaded NPs. Therefore, RT-PCR findings indicated superior apoptotic gene expression via the Bax/Bcl-2, caspase 3, 7, and 9 axes in DTX-loaded hyperbranched MeO-PEG-b-(NIPAAm-co-PBAE) NPs compared to free DTX, which is incongruent with the Annexin-V and cell cycle outcomes. Our findings agree with Sabzi et al. studies reporting the higher amount of cell death after nanoformulation therapy of cancer cells led to upregulations of the Bax, cleaved-caspases of 9,7 and 3 accounting for apoptosis^35^.

### Western blotting used to assess the mitochondrial apoptotic signaling pathways

**Figure 15:**
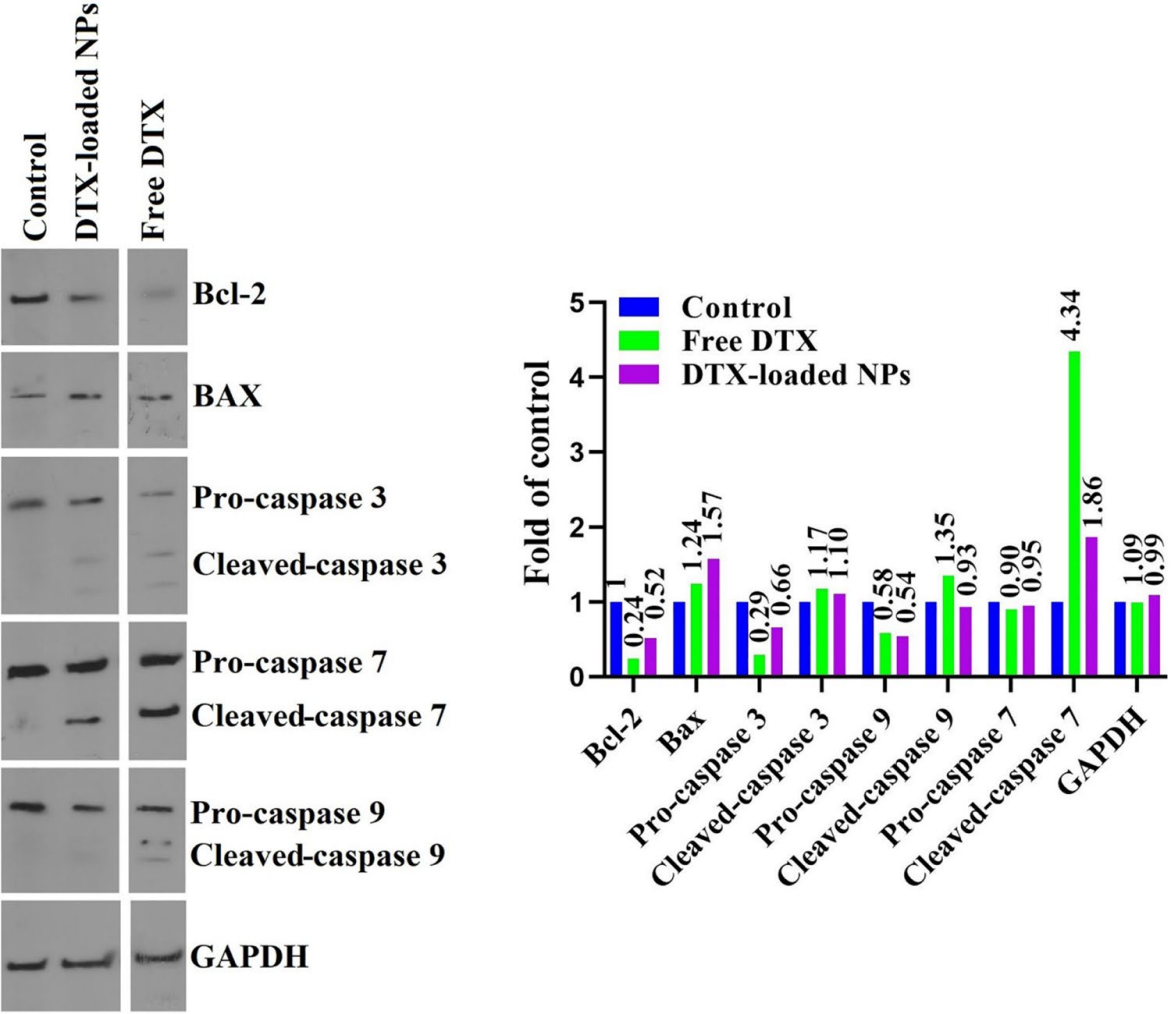
Western blotting images (**A**), and western blotting plot of protein state alterations attributed to the control group (protein state = 1) (**B**) of the MDA-MB-231 cells incubated with free DTX and DTX-loaded NPs with a concentration of IC_50_ dose. Proteins: Bcl-2, Bax, caspases 9, 7, and 3, cleaved-caspases 9, 7, and 3, and GAPDH as internal control, n = 2.

The expression of Bax and Bcl-2, caspase 9, 7, and 3, and cleaved-caspase 9, 7, and 3 in treated MDA-MB-231 cells was evaluated by western immunoblotting as key apoptotic markers after incubation with free DTX and DTX-loaded hyperbranched MeO-PEG-b-(NIPAAm-co-PBAE) NPs to check the mechanisms activity of the caspase-dependent intrinsic apoptosis route. Figure 15 displays the quantitative comparison of protein state with western blotting in MDA-MB-231 cells treated by DTX-loaded NPs and free DTX. Full-length gels and blots are presented in Supplementary Figure S4. Docetaxel triggers the phosphorylation and subsequently inactivates Bcl-2 in apoptosis^58^. Expression of Bax protein as an accelerator of apoptosis to be up-regulated in treated breast cancer cell lines upon DTX-loaded NPs, while anti-apoptotic Bcl-2 expression was down-regulated.

This finding showed higher apoptosis and cell death in cancer cells treated with DTX-loaded NPs because of targeted DTX release from engineered smart NPs based on size shrinkage and charge reversal stimuli to TME. Following the experimental results, the western immunoblotting outcomes exhibited the intrinsic caspase-dependent (mitochondrial) apoptosis pathway in MDA-MB-231 cells incubated with DTX-loaded pH/redox dual-responsive Hb NDDSs.

Safi et al. found that free docetaxel could inhibit MDA-MB-231 breast cancer cell growth by a significant increase in BAX level, as well as a decrease in the levels of Bcl-2 proteins^59^. Dey et al. showed that Western blot analysis induces apoptosis through the Bax, Bcl-2, and caspase 3 proteins in treated MDA-MB-231 cells with alone docetaxel^60^.

## Conclusion

An unprecedented type of pH/redox dual-responsive DTX-loaded hyperbranched MeO-PEG-b-(NIPAAm-co-PBAE) polymeric nanoparticles with charge reversal and size shrinkage behavior was engineered to target TME of breast cancer. By taking advantage of pH and GSH alteration between tumor and normal tissues, size shrinkage and charge reversal NDDSs were designed by incorporating disulfide bonds (S–S) and protonation/deprotonation of amine groups in the structure of highly branched copolymer, which were constructed through atom transfer radical polymerization method, which leads to the specific drug release to the target tissue.

NPs could sustain the resistance of the vehicles during blood circulation, but effortlessly decomposed in TME stimuli can synergistically enhance NPs' accumulation in tumors (~ 100% uptake at 0.5 h) and pH/redox dual-triggered release (~ 100% release at 24 h) of the encapsulated agent in the cytosol of MDA-MB-231 cancer cells, eventuating leading to enriched therapeutic efficacy while decreasing the side effects. The surface charge of NPs changed from negative (-10.6 mV) in a normal physiological condition to positive (+ 7.46 mV) in a leniently acidic tumor microenvironment. The size of NPs shrank from 80.24 ± 13.46 to 32.10 ± 5.9 nm due to the broken down of disulfide bridges stimulated by variations between glutathione (GSH) concentrations in normal and tumor tissues.

In vitro, cytotoxicity experiments consistently verified anti-tumoral properties of DTX loaded-NPs through cell cycle analysis, MTT assay, Annexin V-FITC apoptosis, RT-PCR, and western blotting. The MTT assay showed significantly higher cytotoxicity (*P*_value_ < 0.0001). For DTX-loaded NPs compared to free DTX on the MDA-MB-231 cells. Cell cycle results exhibited that DTX-loaded NPs arrested cells in the S and G2/M phases. Annexin V assay results exhibited a significantly higher amount of induction of apoptotic in the MDA-MB-231 cancer cell line treated with DTX-loaded NPs (more than 71.5 ± 2.8%) in comparison to the free DTX (42.34 ± 3.1%) (*P*_value_ < 0.001). Also, gene-level RT-PCR and protein-level western-blotting demonstrate superior induction of tumor cell apoptosis via Bax/Bcl-2, caspase 3, 7, and 9, dependent intrinsic mitochondrial apoptosis pathway. The results indicated that the new architectural pH and redox dual-responsive self-assemble hyperbranched copolymer with DTX-loaded NDDSs with TME-responsive size shrinkage and charge reversal ability showed great potential in targeted delivery of DTX to tumor tissue.

### Supplementary Information

Supplementary Information.

## Data Availability

All data generated or analyzed during this study are included in this published article.
