# Supplementary material for: Dual pH/redox-responsive hyperbranched polymeric nanocarriers with TME-trigger size shrinkage and charge reversible ability for amplified chemotherapy of breast cancer
Source: Sci Rep. 2024 Apr 12;14:8567. doi: 10.1038/s41598-024-57296-4 (PMC11349913; doi:10.1038/s41598-024-57296-4)
Supplement: Supplementary file 1 — Supplementary Information. [file 41598_2024_57296_MOESM1_ESM.docx]

**Supplementary Information**

**Dual pH/Redox-Responsive Hyperbranched Polymeric Nanocarriers with TME-Trigger Size**

**Shrinkage and Charge Reversible Ability for Amplified Chemotherapy of Breast Cancer**

Fahimeh Badparvar^1^, Ahmad Poursattar Marjani^1,^*, Roya Salehi^2,^*, and Fatemeh Ramezani^3^

*^1^Department of Organic Chemistry, Faculty of Chemistry, Urmia University, Urmia, Iran*

*^2^Drug Applied Research Center and Department of Medical Nanotechnology, Faculty of Advanced Medical Sciences, Tabriz University of Medical Sciences, Tabriz, Iran*

*^3^Department of Medical Nanotechnology, School of Advanced Medical Sciences, Tabriz*

*University of Medical Sciences, Tabriz, Iran*

* E-mail: [a.poursattar@urmia.ac.ir](mailto:a.poursattar@urmia.ac.ir); [salehiro@tbzmed.ac.ir](mailto:salehiro@tbzmed.ac.ir)

**Infrared Spectroscopy Study.** The chemical functional groups present in, 2-mercaptoethanol, BHES, DSDA, acrylate-terminated poly(β-amino ester)s, MeO-PEG, bromine-terminal MeO-PEG macroinitiator, NIPAAm, hyperbranched MeO-PEG-β-(NIPAAm-co-PBAE) were studied via FTIR spectroscopy (Figure S1).

According to Figure S1A, the absorption band in the broad frequency range 3200–3501 cm^-1^ is due to the –OH groups of 2-mercaptoethanol. The around 2878–2950 cm^-1^ peak is relevant to the aliphatic C–H stretching. Also, the –SH stretching of the 2-mercaptoethanol appeared at 2562 cm^-1^, and the C–S bending appeared at 938 cm^-1^.

Figure S1B shows the spectrum of the BHES. Two apparent differences in observed bands with pure 2-mercaptoethanol suggest that the oxidation of 2-mercaptoethanol was done. The first difference in the disappearance of peak frequency at 2562 cm^-1^ corresponds to the –SH groups. The S-S band pick has appeared at 592 cm^-1^.

In the case of Figure S1C, the spectrum of the DSDA was investigated by FT-IR spectroscopy. The disappearance of the –OH peak (3350 cm^-1^) and the appearance of two peaks for C=O (1729 cm^-1^) and C=C (1631 cm^-1^) groups are robust evidence for successful synthesizes of DSDA.

Figure S1D illustrates the spectrum of the acrylate-terminated poly(β-amino ester)s containing disulfide bond as cross-linker (PBAE). The form of a broad absorbance band at the area of 3700–2500 cm^−1^ was a property of OH stretching, the new stretching peak of the ester bond carbonyl group appeared at wave number 1730 cm^−1^, and the bands at 1188 and 1052 cm^−1^ were attributed to CO ester bonds. The 2858 and 2952 cm^−1^ peaks demonstrated CH_3_ and CH_2_ symmetric and asymmetric aliphatic stretching vibration. The absorption stretching vibration peak of vinyl (C=C) has appeared at 1632 cm^−1^. This peak confirmed that the acrylate-terminated functional group was synthesized at the end of PBAE.

Figures S1E and S1F show the spectrums of the pure MeO-PEG and bromine-terminal MeO-PEG macroinitiator; the peak in 524 cm^-1^ is related to the C–Br group and the stretching vibration absorption peak of the acyl group’s carbonyl appeared at 1729 cm^-1^. This comparison has confirmed of bromine-terminal MeO-PEG macroinitiator.

Figure S1G shows the spectrum of the pure NIPAAm. Notable peaks appear at 3287 cm^-1^ for stretching vibration of N–H, 2969, and 2926 cm^-1^ for asymmetric and symmetric -CH groups, respectively. The absorption stretching vibration C=O amide groups peak is presented at 1654 cm^-1^. 1542 cm^-1^ band can be assigned to N–H bending vibration of NIPAAm. The stretching vibration peak at 1366 cm^-1^ is called C–N band. The peak related to bending vibrations of –CH_2_ and –CH_3_ appeared at 1460 and 1386 cm^-1^. The stretching vibration of =C-H groups is presented at 3063 cm^-11^.

Figure S1H shows the spectrum of the hyperbranched MeO-PEG-β-(NIPAAm-co-PBAE) copolymer. In the ranges, at wave number 1632 cm^−1^, the absorption peak of the vinyl bond disappeared, which refers to the ATRP polymer synthesized from terminal acrylate PBAE as the monomer. Also, the carbonyl bond of macromonomers appeared at 1729 cm^−1^, and the sharp stretching vibration aliphatic group peak at 2921 cm^−1^ was referred to the methyl group of PEG backbone as macroinitiator, then added by ATRP reaction. Based on FTIR results, the formation of a hyperbranched MeO-PEG-β-(NIPAAm-co-PBAE) copolymer was successful.


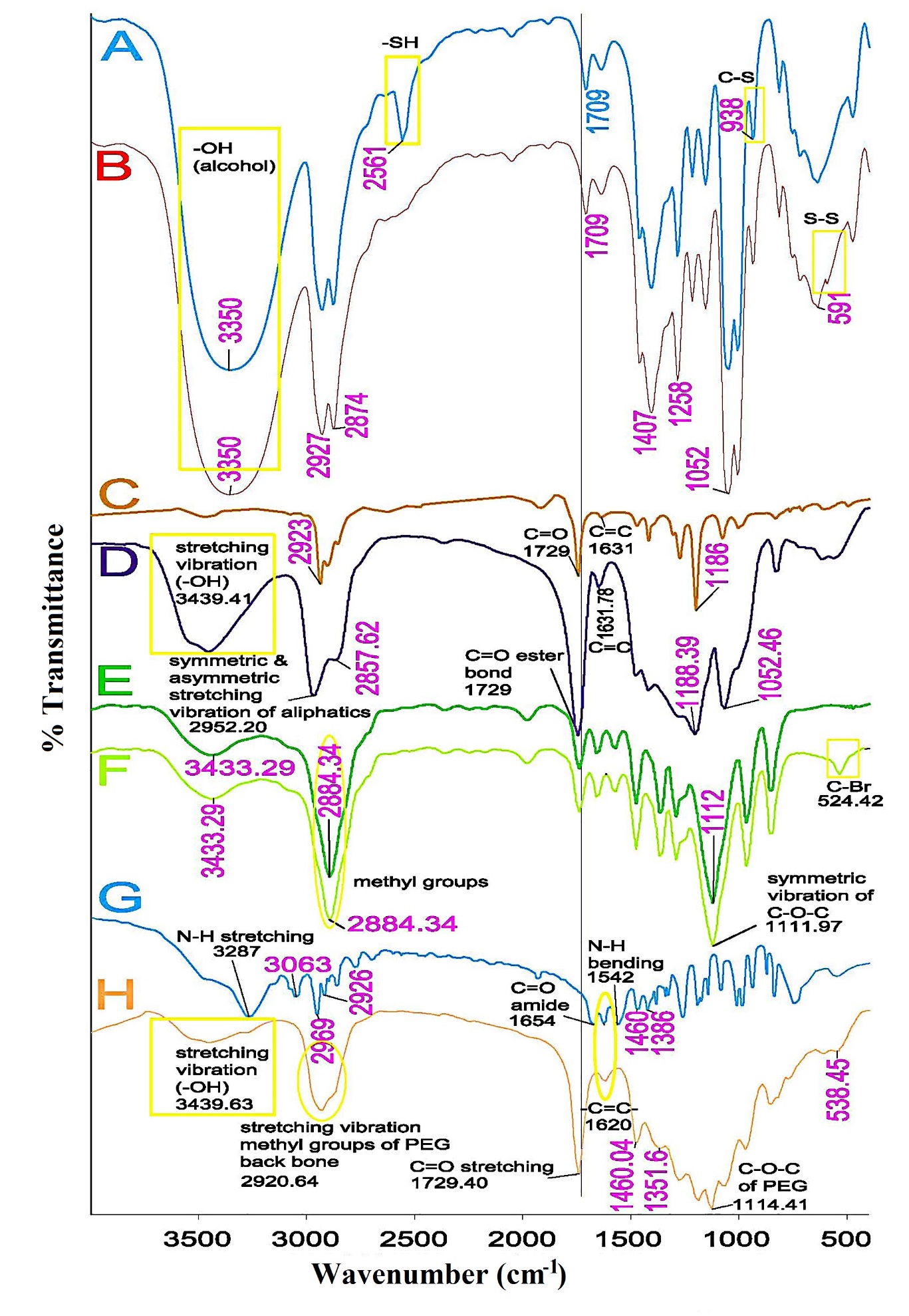


**Figure S1.** FTIR spectra of 2-mercaptoethanol (**A**), bis(2-hydroxyethyl) disulfide (BHES) (**B**), 2,2'-dithiodiethanol diacrylate (DSDA) (**C**), acrylate-terminated poly (β-amino ester)s (**D**), pure MeO-PEG (**E**), bromine-terminal MeO-PEG macroinitiator (MeO-PEG-Br) (**F**), pure NIPAAm (**G**), and hyperbranched MeO-PEG-β-(NIPAAm-co-PBAE) copolymer (**H**)

**^1^H-NMR analysis.** The ^1^H-NMR spectra related to the synthesized BHES (Figure S2A), 2,2'-dithiodiethanol diacrylate (DSDA) (Figure S2B), acrylate-terminated poly (ß-amino ester) (Figure S2C), MeO-PEG-Br macroinitiator (Figure S2D) were represented in Figure S2.

According to Figure S2A, related to BHES (δ (ppm)), the presented peaks at 2.8 (t, HOCH_2_**CH_2_**SS**CH_2_**CH_2_OH), 3.8 (t, HO**CH_2_**CH_2_SSCH_2_**CH_2_**OH), 3.4 (s, (**H**OCH_2_CH_2_SSCH_2_CH_2_O**H**) confirmed the correctness of the BHES structure.

The ^1^H-NMR spectra of 2,2'-dithiodiethanol diacrylate (DSDA) was presented in Figure S2B. The peaks at 4.3 ppm, and 2.9 ppm were related to (–**CH_2_**CH_2_SSCH_2_**CH_2_**–), and (**CH_2_**SS**CH_2_**). The appearance of the peaks related to the acrylate group at 5.5 and 6.3 ppm (C**H_2_**CHCOO–) and 6.08 ppm (CH_2_C**H**COO–), as well as the removal of the hydroxyl group peak, is a strong evidence for the confirmation of the DSDA synthesizes.

In the ^1^H-NMR spectra of the acrylate-terminated poly(β-amino ester) Figure S2C polymer characteristic peaks appeared at (δ (ppm)): 1.1–1.4 (m, –NCH_2_C**H_2_**CH_2_OH), 1.60 (s, C**H_2_**CHCOOCH_2_C**H_2_**–, and –C**H_2_**CH_2_OOCCH_2_CH_2_N–), 2.3–2.4 (m, –NC**H_2_**(CH_2_)_2_OH and –COOC**H_2_**CH_2_N–), 2.71–2.74 (m, –NC**H_2_**CH_2_OOC–), 2.88 (m, –O-CH_2_C**H_2_**-SS-C**H_2_**CH_2_-O-, (cystamine core)), 3.6 (s, –N(CH_2_)_2_C**H_2_**OH), 4.0 (s, –CH_2_C**H_2_**OOCCH_2_CH_2_N–), 4.4 (t, -O-C**H_2_**CH_2_-SS-CH_2_C**H_2_**-O-, (cystamine core)), 5.28 (s, –N(CH_2_)_3_O**H**), 5.7 (m, C**H_2_**CHCOOCH_2_CH_2_– (terminal acrylate)), 6.07 (m, CH_2_C**H**COOCH_2_CH_2_–(terminal acrylate)), 6.3 (m, C**H_2_**CHCOOCH_2_CH_2_–(terminal acrylate)).

The above interpretations confirm the successful preparation of acrylate-terminated poly (ß-amino ester) polymer containing the disulfide bond as a reduction section and ester linkages as a biodegradable section.

The average molecular weight of acrylate-terminated PBAE was obtained 2493 g/mol from Equations S1 and S2 with the aid of integrating of the peaks in ^1^H-NMR spectrum.

$\frac{peak area of repeating units}{\# of protons in repeating units}=$*Integral per proton* (S1)

*M_n_ = (M_wt_ of repeating Unit) n + (M_wt_ of end groups)* (S2)

The ^1^H-NMR of the MeO-PEG-Br macroinitiator is presented in Figure S2D. After the esterification, a new resonance signal, including protons of -COC(C**H_3_**)_2_Br initiating group, is located at 1.93 ppm in the ^1^H-NMR spectrum. Respectively, typical resonance signals of the PEG chain appeared at (δ (ppm)): 3.6 (-OC**H_2_**C**H_2_**), 3.26 (-OC**H_3_**). So, according to ^1^H-NMR spectra, the successful introduction of the ATRP initiating group was demonstrated.

The molar mass of MeO-PEG-Br macroinitiator was obtained 5153 g/mol by mass of repeated unit group (-OCH_2_CH_2_)_113_ and end-groups of (-CH_3_) and (-OCOC(CH**_3_**)_2_Br) according to Equation S2.

^1^H-NMR of final MeO-PEG-β-(NIPAAm-co-PBAE) copolymer is represented in Figure 2. The dominant resonances were observed at (δ (ppm)): 1.1 (-CH(C**H_3_**)_2_ (NIPAAm)), 1.2 (–NCH_2_C**H_2_**CH_2_OH (PBAE)), 1.4 (-C**H_2_**-CHCO(NIPAAm)), 1.68 (–N(CH_2_)_2_COOCH_2_C**H_2_**C**H_2_**– and C**H_2_**C**H_2_**CH_2_COOCH_2_C**H_2_**C**H_2_**–), 1.43 (-CO(CH)-(C**H_2_**)_2_(NIPAAm)), 1.98 (-C**H_3_**(PEG)), 2.4–2.5 (–NC**H_2_**(CH_2_)_2_OH and –COOC**H_2_**CH_2_N–(PBAE)), 2.7 (–COOCH_2_C**H_2_**N–(PBAE)), 3.24 (-OC**H_3_**(PEG)), 3.6 (-OC**H_2_**C**H_2_**(PEG)), 3.8 (-CO-C**H**-(CH_3_)_2_ (NIPAAm)), 4.0 (–N(CH_2_)_2_COOC**H_2_**CH_2_– (PBAE)).

The proton signals at δ = 5.0 to 6.5 ppm due to the acrylate groups of PBAEs and NIPAAm monomers wholly disappearing, indicating the complete elimination of monomers during the ATRP polymerization. Also, the appearance of new peaks at 3.8 (CH(C**H_3_**)_2_) and 1.1 (C**H**(CH_3_)_2_) confirmed the introduction of NIPAAm in copolymer structure.

The above results proved the successful synthesis of hyperbranched MeO-PEG-β-(NIPAAm-co-PBAE) copolymer through the ATRP reaction.

The molecular weight of Hb MeO-PEG-β-(NIPAAm-co-PBAE) copolymer using Equation S3 and S4 based on the ^1^H-NMR spectra was obtained 18967 g/mol, and the calculated ratio of 27.12, 52.57, and 20.25 %W for PEG, PBAE, and NIPAAm, were determined respectively.

*n _Polymer_* = $\frac{\sum_{i=1}^{m} lᵢ}{pᵢ}$ (S3)

*% Weight_i_* = $\frac{nᵢ \times Mᴡᵢ}{\sum_{i=1}^{m} nᵢ \times Mᴡᵢ} \times100$ (S4)

Where "p_i_" and "l_i_" are the number and integration of protons that related to i_th_ signal of copolymer, and "m" is the number of copolymer peaks.


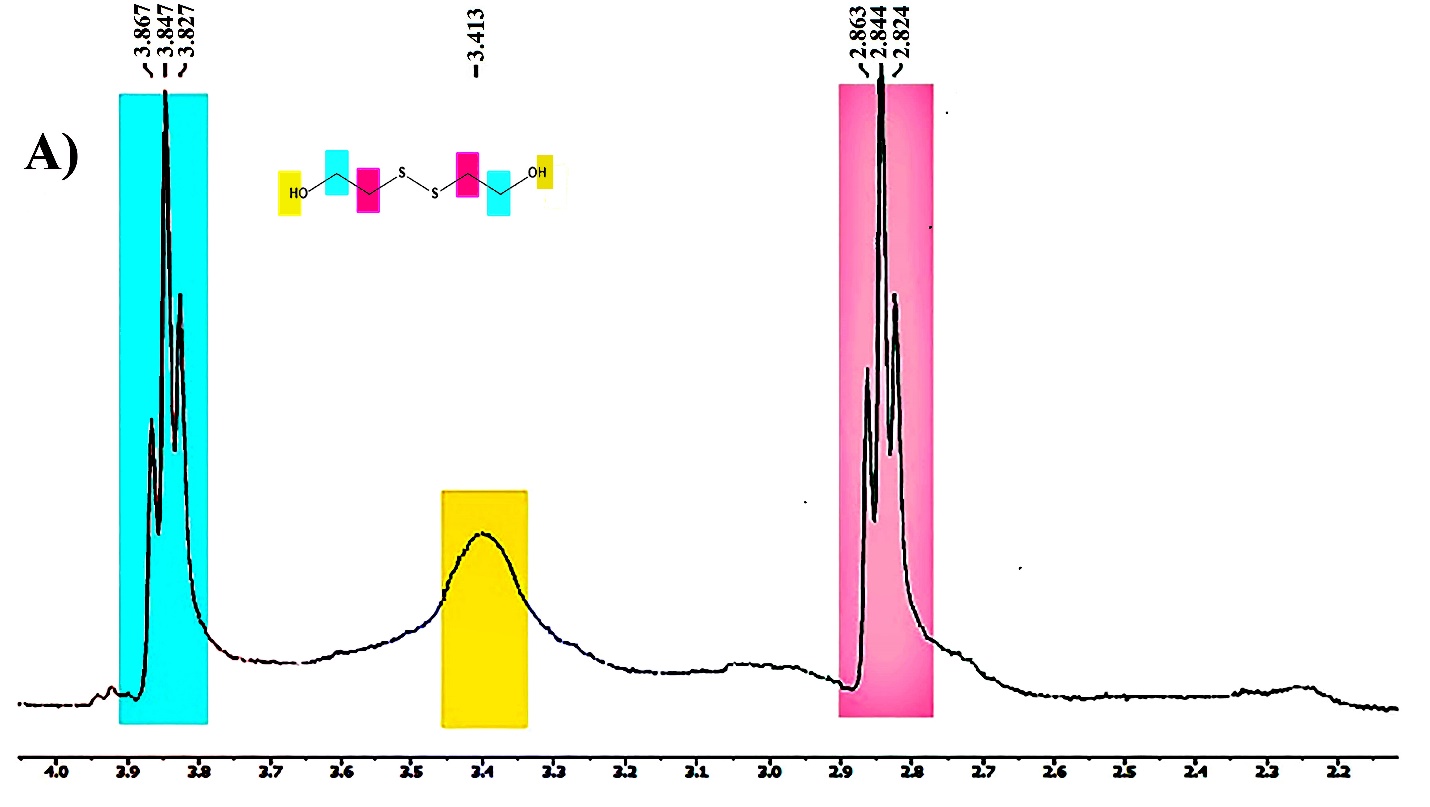


**
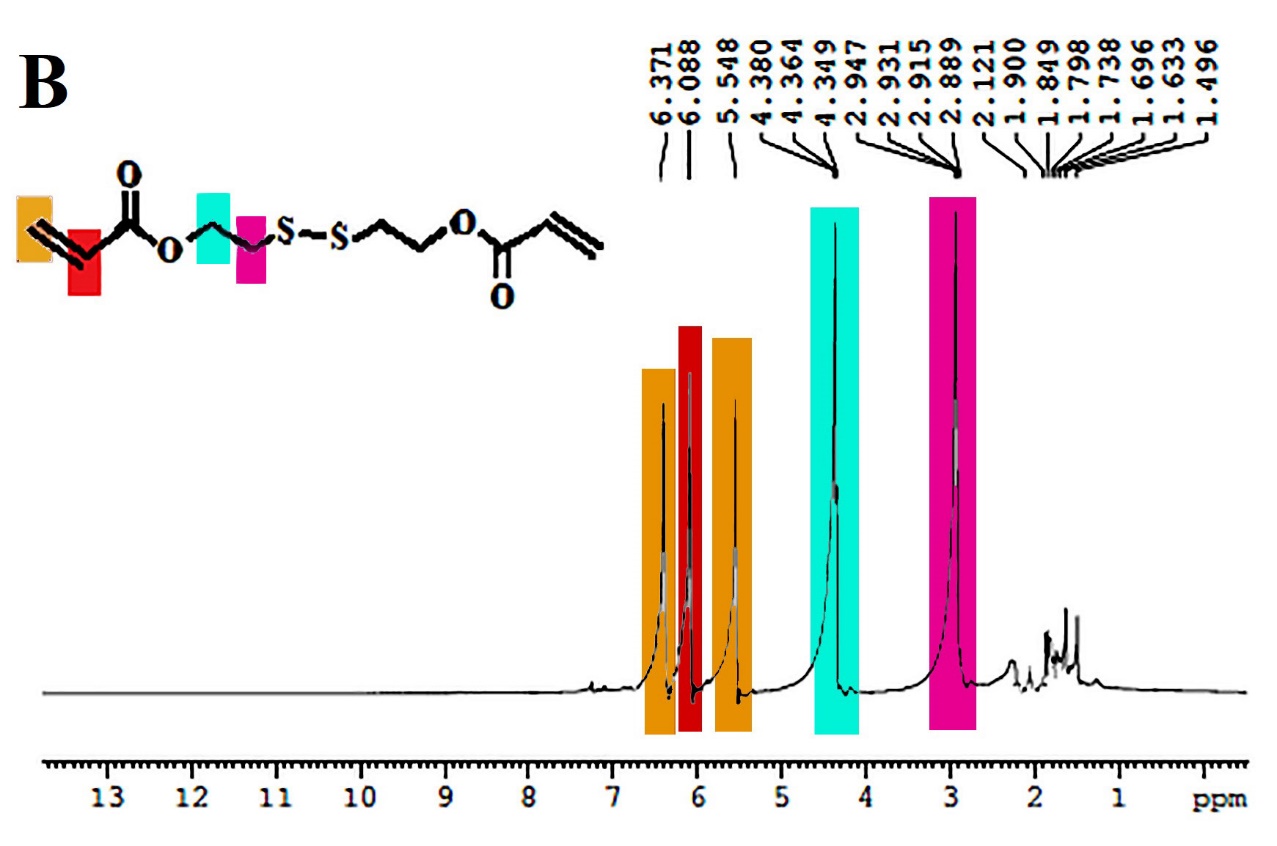
**

**
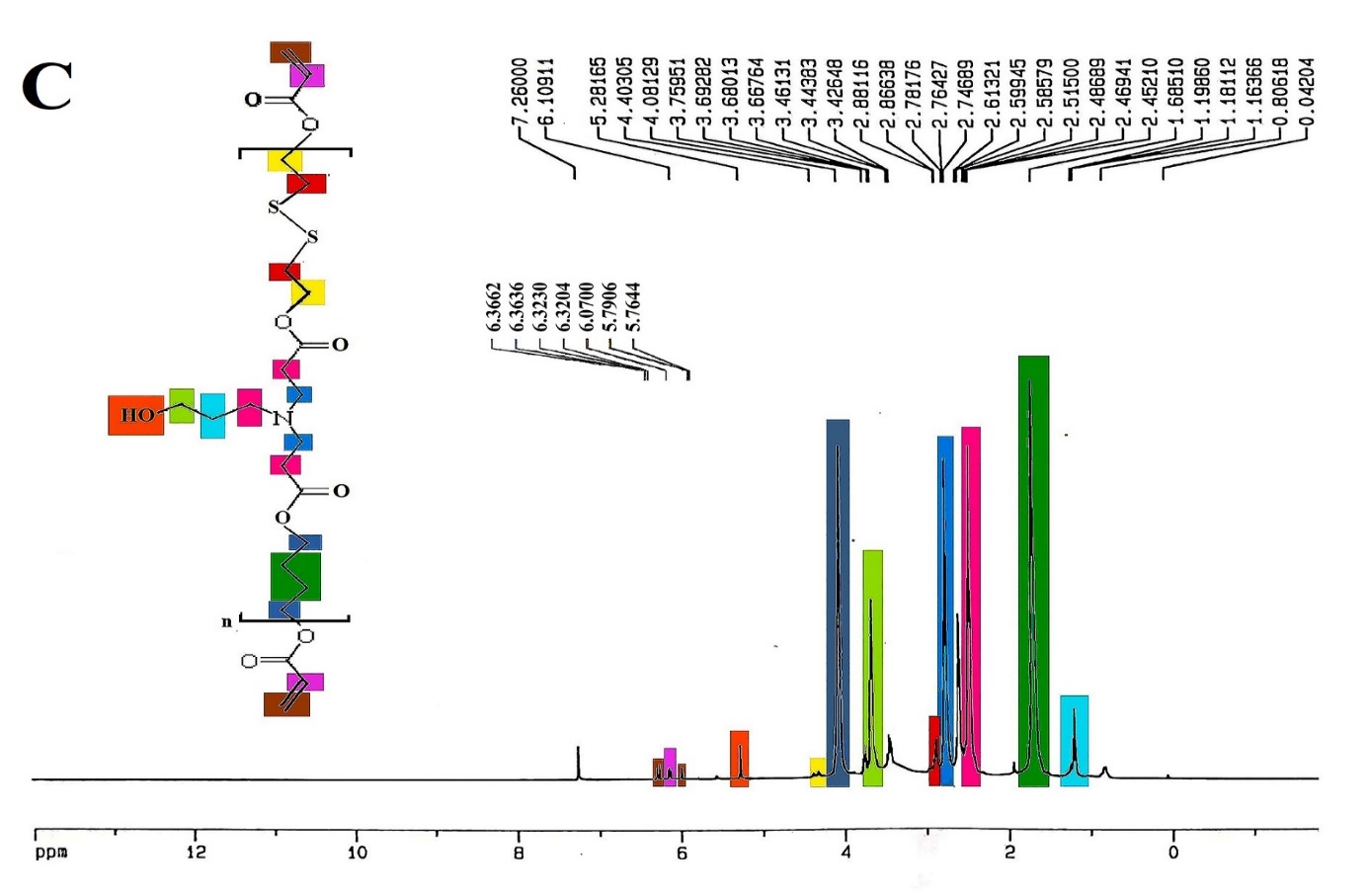
**

**
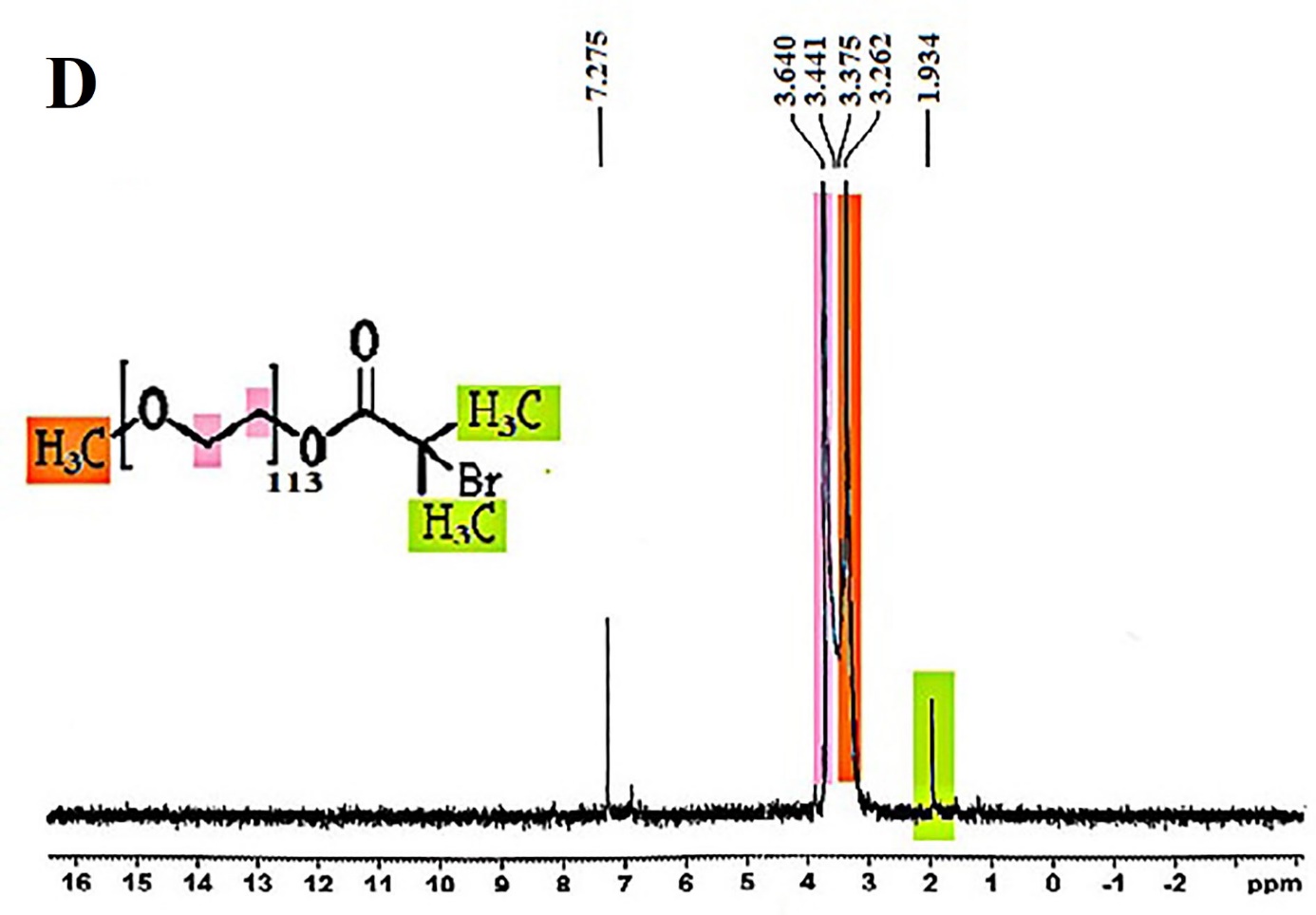
**

**Figure S2.** The ^1^H-NMR spectra of bis(2-hydroxyethyl) disulfide (BHES) (**A**), 2,2'-dithiodiethanol diacrylate (DSDA) (**B**), acrylate-terminated poly (ß-amino ester) (**C**), bromine-terminal MeO-PEG macroinitiator (MeO-PEG-Br) (**D**).

**Calculation of Copolymer Molar Mass and weight Percentage Using Data of ^1^H-NMR Spectra.**

The number average molecular weight (Mn) could be determined by end-group and repeating units analysis using ^1^H-NMR spectra based on Equations S2 and S5.

Integral per proton calculated with the vinyl end-group proton signals (δ = 6.3 ppm, (1H), (C**H_2_**CH-), integral = 0.12, and δ = 6 ppm, (1H), (C**H_2_**CH-), integral = 0.12; δ = 5.7 ppm, (1H), (CH_2_C**H**-), integral = 0.1) following equation S5.

Integral per proton = $\frac{Sum of end group proton integrals}{\# of protons in the end groups}$ (S5)

$$\frac{0.1+0.12+0.12}{6}=0.057 per proton$$

The degree of polymerization of repeating unit signals (δ = 1.1–1.4 ppm, (2H), (–NCH_2_C**H_2_**CH_2_OH), integral = 0.2; δ = 1.6 ppm, (4H), (C**H_2_**CHCOOCH_2_C**H_2_**–), integral = 3.4; δ = 2.3-2.4 ppm, (4H), (–NC**H_2_**(CH_2_)_2_OH and –COOC**H_2_**CH_2_N–), integral = 2; δ = 271-2.74 ppm, (4H), (–NC**H_2_**CH_2_OOC–), integral = 2; δ = 2.88 ppm, (4H), (–O-CH_2_C**H_2_**-SS-C**H_2_**CH_2_-O-), integral = 0.01; δ = 3.6 ppm, (2H) , (–N(CH_2_)_2_C**H_2_**OH), integral = 0.2; δ = 4.0 ppm, (4H), –CH_2_C**H_2_**OOCCH_2_CH_2_N–), integral = 2; δ = 4.4 ppm, (4H), (-O-C**H_2_**CH_2_-SS-CH_2_C**H_2_**-O-), integral = 0.01; δ = 5.28 ppm, (1H), (–N(CH_2_)_3_O**H**), integral = 0.01) based on Equation S1.

$\frac{0.2+3.4+2+1+2+0.2+2+0.01+0.08+0.01}{27n}=0.057$ n= 7.08 ~7

The molecular mass (M_n_) of acrylate-terminated PBAE was obtained 2493 g/mol from calculations investigated from Equation S2 with the aid of integrating of the peaks in ^1^H-NMR spectrum.

M_n_ = $7\times\left( 346 \right)+71=2493 g/mol$

The molecular mass (M_n_) of MeO-PEG-Br macroinitiator was calculated from Equation S2 ,mass of repeated unit group (-OCH_2_CH_2_)_113_ and end-groups of (-CH_3_) and (-OCOC(CH**_3_**)_2_Br).

M_n_ = (113 $\times$ (44))+ 181 = 5153 g/mol

Based on the integration value of –CH_3_ (A≈ 0.083) peak of PEG at δ = 1.98 ppm, integral per proton was obtained equal to 0.0138. Then, the other peaks integration amount was divided by this integration value.

NIPAAM: integration value at δ = 3.8 ppm (–CH(CH_3_)_2_ peak), 1H for one group per molecule, was 0.466 that is divided by 0.0138 (integral per proton). Then, the result (33.76 ≈ 34), was used for determining relative mole of NIPAAM monomer in copolymer, according Equation S3.

n _NIPAAM_ = (34/1)/1 = 34

PBAE: Integration value at δ = 2.7 ppm (–COOCH_2_C**H_2_**N– peak), 4H for 7 group per molecule, was 1.38 that is divided by 0.0138 (integral per proton). Then, the result (100), was used for determining mole of PBAE monomer in copolymer, according Equation S3:

n _PBAE_ = (94.20/28)/1 = 3.57 ≈ 4

At last, considering obtained mole of copolymer sections, the copolymer molar mass is calculated according to Equation S2:

M_n_ = (1$\times5153$) + (4$\times2493$) + (34$\times113$) = 18967 g/mol

The weight percentages of PEG, PBAE, and NIPAAM based on ^1^H-NMR spectrum was calculated according to Equation S4:

% PEG =$\frac{1 \times5153}{1 \times5153 +4 \times2493 + 34 \times113}$ $\times100$= 27.12 %

% PBAE =$\frac{4 \times2493}{1 \times5153 +4 \times2493 + 34 \times113}$ $\times100$= 52.57 %

% NIPAAM =$\frac{34 \times113}{1 \times5153 +4 \times2493 +34 \times113}$ $\times100$= 20.25 %

**Table S1.** Evaluation and optimization of drug loading capacity and encapsulation efficiency.

| Ratio of NPs:Drug (w/w) | Encapsulation efficiency (DEE%) | Drug loading content (DLC%) |
| --- | --- | --- |
| 2:1 | *36.5* $\pm$ *6.23* | *1*$.36 \pm$ *0.07* |
| 5:1 | *54.7*$\pm$ *4.63* | $2.19 \pm$ *1.09* |
| 10:1 | *98.3*$\pm1.11$ | *9.83*$\pm$ *1.02* |

**
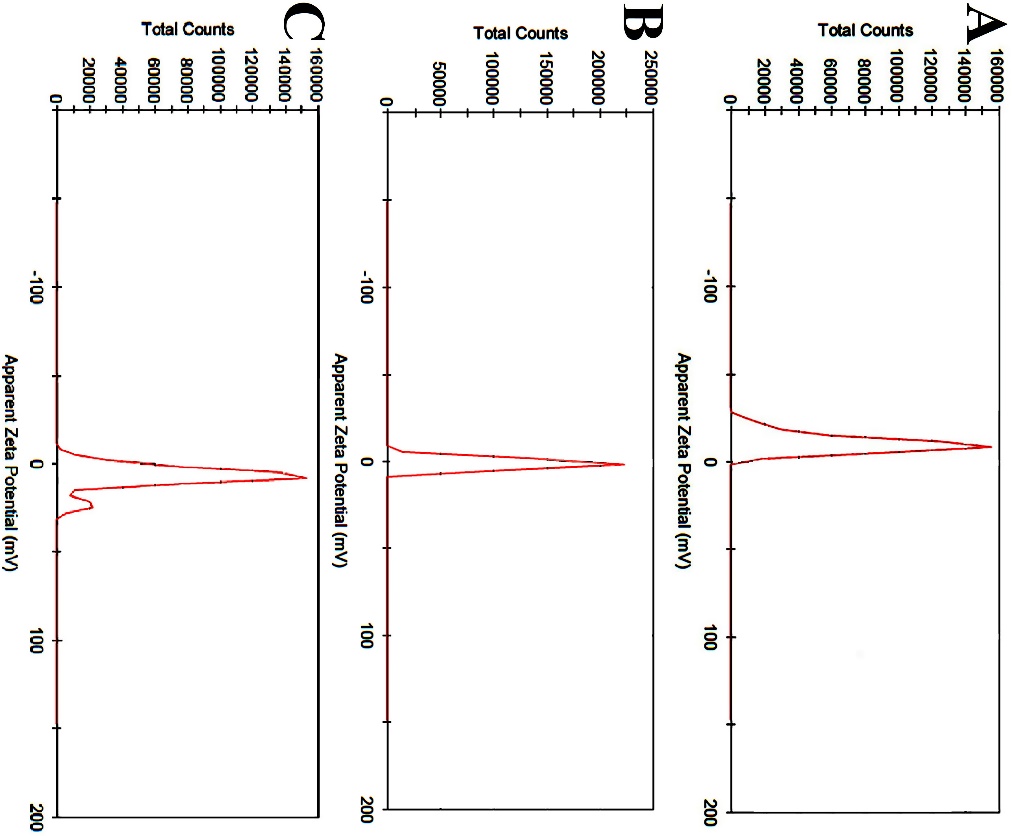
**

**Figure S3.** The zeta potential of blank MeO-PEG-b-( NIPAAm-co-PBAE) NPs (**A**), Drug-loaded NPs in PBS (pH = 7.4) (**B**); And the zeta potential of blank NPs after 4 h incubation under weakly acidic conditions (PBS, pH = 6.4) (**C**).

**Western blotting.** Western blot analysis was performed according mentioned protocol in the manuscript. All bands were obtained from same gel after some stripping and reprobing. Figure S5 showed the western blot original images presenting GAPDH (**A**), pro- and cleaved-caspase 7 (**B**), pro- and cleaved-caspase 3 (**C**), pro- and cleaved-caspase 9 (**D**), Bcl-2 (**E**), Bax (**F**). There are 3 bands in each original scanned image that were delineated in GAPDH image as follow: C = Control group, DEP = hyperbranched MeO-PEG-b-(NIPAAm-co-PBAE) copolymeric nanocarrier, DTX = Docetaxel (DTX). The images were cut on account of their lack of relevance to the subject matter under investigation.


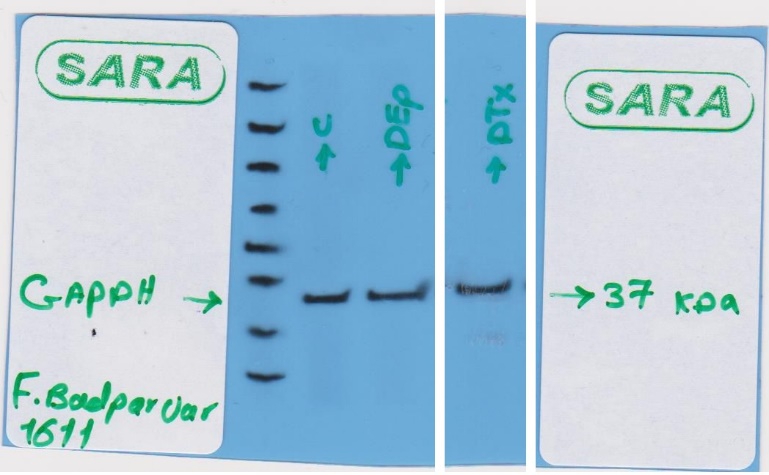


**Figure S4 (A).** Original western blot image of GAPDH. C = Control group, DEP = DTX-loaded MeO-PEG-b-(NIPAAm-co-PBAE) copolymeric nanocarrier, DTX = Docetaxel.


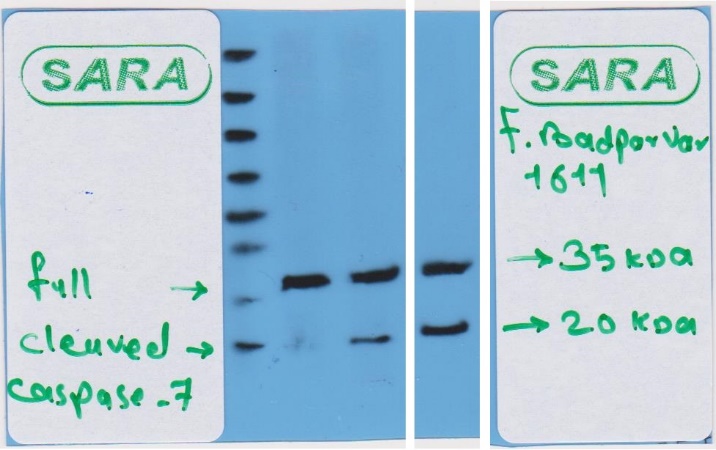


**Figure S4 (B).** Western blot original image for pro- and cleaved-caspase 7. The label of “full” means is “pro-caspase 7”.


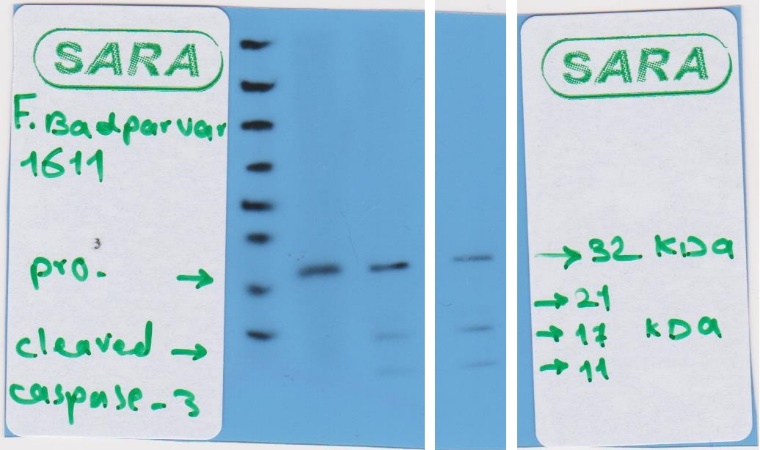


**Figure S4 (C).** Western blot original image for pro- and cleaved-caspase 3.


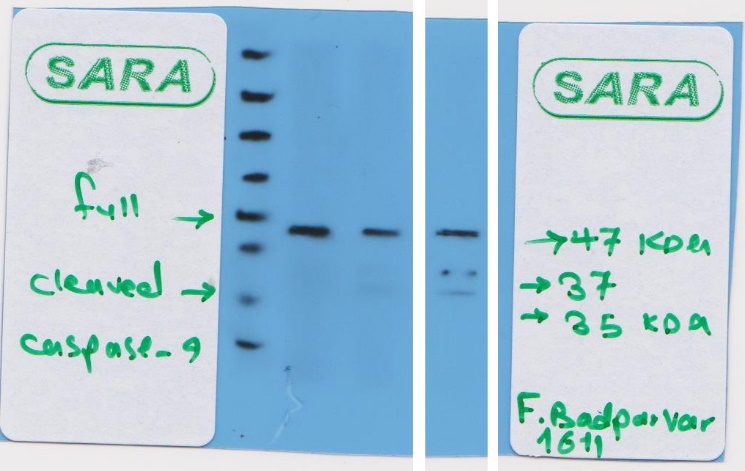


**Figure S4 (D).** Western blot original image for pro- and cleaved-caspase-9. The label of “full” means is “pro-caspase 9”.

**
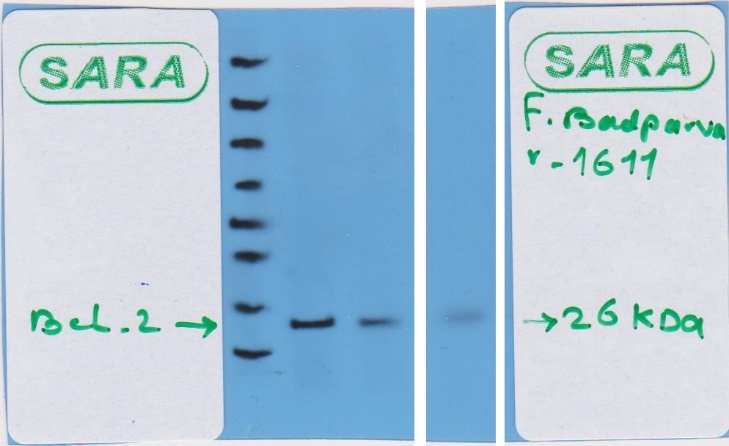
**

**Figure S4 (E).** Western blot original image for Bcl-2.


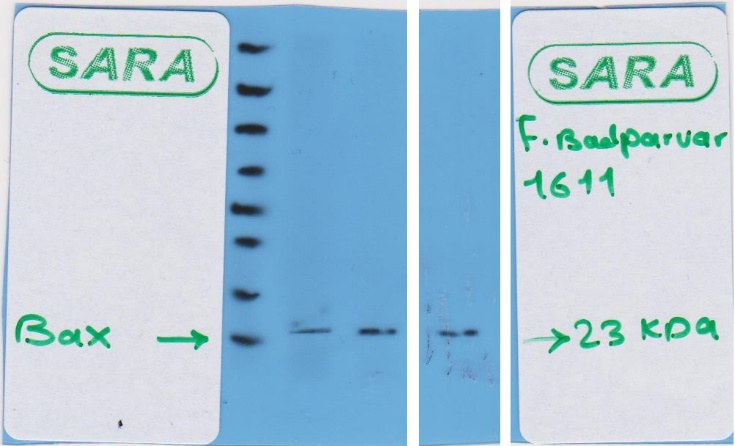


**Figure S4 (F).** Western blot original image for Bax.

1 Kim, M. H., Kim, J.-C., Lee, H. Y., Dai Kim, J. & Yang, J. H. Release property of temperature-sensitive alginate beads containing poly (N-isopropylacrylamide). Colloids Surf. B: Biointerfaces **46**, 57–61 (2005).
